# The role of case proximity in transmission of visceral leishmaniasis in a highly endemic village in Bangladesh

**DOI:** 10.1371/journal.pntd.0006453

**Published:** 2018-10-08

**Authors:** Lloyd A. C. Chapman, Chris P. Jewell, Simon E. F. Spencer, Lorenzo Pellis, Samik Datta, Rajib Chowdhury, Caryn Bern, Graham F. Medley, T. Déirdre Hollingsworth

**Affiliations:** 1 Zeeman Institute, University of Warwick, Coventry, UK; 2 School of Life Sciences, University of Warwick, Coventry, UK; 3 Centre for Mathematical Modelling of Infectious Diseases, London School of Hygiene and Tropical Medicine, London, United Kingdom; 4 Centre for Health Informatics, Computing And Statistics, Lancaster University, Lancaster, UK; 5 Department of Statistics, University of Warwick, Coventry, UK; 6 National Institute of Water and Atmospheric Research, Wellington, New Zealand; 7 National Institute of Preventive and Social Medicine (NIPSOM), Mohakhali, Dhaka, Bangladesh; 8 International Centre for Diarrhoeal Disease Research, Bangladesh (icddr,b), Dhaka, Bangladesh; 9 Department of Epidemiology and Biostatistics, University of California San Francisco, San Francisco, California, USA; 10 Big Data Institute, Li Ka Shing Centre for Health Information and Discovery, University of Oxford, Oxford, UK; Genesis Laboratories, Inc., UNITED STATES

## Abstract

**Background:**

Visceral leishmaniasis (VL) is characterised by a high degree of spatial clustering at all scales, and this feature remains even with successful control measures. VL is targeted for elimination as a public health problem in the Indian subcontinent by 2020, and incidence has been falling rapidly since 2011. Current control is based on early diagnosis and treatment of clinical cases, and blanket indoor residual spraying of insecticide (IRS) in endemic villages to kill the sandfly vectors. Spatially targeting active case detection and/or IRS to higher risk areas would greatly reduce costs of control, but its effectiveness as a control strategy is unknown. The effectiveness depends on two key unknowns: how quickly transmission risk decreases with distance from a VL case and how much asymptomatically infected individuals contribute to transmission.

**Methodology/Principal findings:**

To estimate these key parameters, a spatiotemporal transmission model for VL was developed and fitted to geo-located epidemiological data on 2494 individuals from a highly endemic village in Mymensingh, Bangladesh. A Bayesian inference framework that could account for the unknown infection times of the VL cases, and missing symptom onset and recovery times, was developed to perform the parameter estimation. The parameter estimates obtained suggest that, in a highly endemic setting, VL risk decreases relatively quickly with distance from a case—halving within 90m—and that VL cases contribute significantly more to transmission than asymptomatic individuals.

**Conclusions/Significance:**

These results suggest that spatially-targeted interventions may be effective for limiting transmission. However, the extent to which spatial transmission patterns and the asymptomatic contribution vary with VL endemicity and over time is uncertain. In any event, interventions would need to be performed promptly and in a large radius (≥300m) around a new case to reduce transmission risk.

## Author summary

Visceral leishmaniasis (VL), a fatal parasitic disease transmitted by sandflies, has been targeted for elimination as a public health problem in the Indian subcontinent by 2020. The goal has been reached in the majority of endemic regions in Bangladesh, India and Nepal, but the disease persists in several hotspots. Better understanding of spatial clustering of VL cases and the role of asymptomatically infected individuals in transmission is required to improve control interventions and sustain the elimination target. To address this issue, we have fitted an individual-level spatiotemporal model of VL transmission to geo-located incidence data from Bangladesh to estimate the rate at which VL risk decreases with distance from a case and the potential contribution of asymptomatic individuals to transmission. Our results suggest that VL risk decreases quickly with distance and that symptomatic individuals are the main drivers of transmission, highlighting the potential for spatially-targeted control interventions to reduce transmission.

## Introduction

Visceral leishmaniasis (VL), the world’s second most lethal vector-borne parasitic disease, has been targeted for elimination as a public health problem in the Indian subcontinent (ISC) by 2020 [1]. The target is an incidence of less than 1 VL case/10,000 people/year at subdistrict level in Bangladesh and India and district level in Nepal. The reported annual number of cases in the ISC has decreased by approximately 80% since 2011 (from ∼37,000 to ∼6,750) [2], and as of 2017 the target had been reached in all subdistricts in Bangladesh, in approximately 85% of endemic subdistricts in India, and in all districts in Nepal for the previous 4 years [3–7]. Bangladesh has seen a more than 90% reduction in VL incidence since 2011 (from 2874 cases in 2011 to just 255 in 2016) [2], following a large epidemic wave lasting over a decade [8, 9]. The district that has consistently had the highest incidence is Mymensingh, within which Fulbaria (the source of the data analysed in this study) and Trishal have been the most endemic subdistricts and the last to reach the elimination target [9]. Despite the overall decline in incidence in the ISC, several subdistricts that have not had cases for a number of years have reported new cases in 2016 and 2017 [7, 10, 11], highlighting the need to maintain surveillance and control efforts as the target is approached and address gaps in understanding of VL transmission. Two key knowledge gaps are understanding of spatial variation in transmission and the role of asymptomatically infected individuals (individuals infected with *Leishmania donovani* who do not develop clinical symptoms) in transmission [4, 12–14].

Asymptomatic individuals significantly outnumber clinical VL patients in the ISC, with estimates of the asymptomatic-to-symptomatic incidence rate ratio varying from 4:1 [15] to 17:1 [4, 16]. Parasites have been demonstrated in the blood smears of asymptomatic individuals [17] and modelling has suggested that asymptomatic individuals could be the main drivers of transmission if they are infectious to sandflies [18–20]. Evidence of ongoing transmission, in the form of sporadic outbreaks, in regions in Nepal and Bhutan where there has historically been little symptomatic disease but asymptomatic individuals are present [21, 22] suggests that asymptomatic individuals can infect sandflies and contribute to transmission.

In recent xenodiagnosis experiments, asymptomatic individuals (defined by high anti-leishmanial antibody titres by the direct agglutination test (DAT) or rK39 enzyme-linked immunosorbent assay (ELISA) and no symptoms or prior history of VL) failed to infect sandflies fed upon them, whereas clinical VL cases and post kala-azar dermal leishmaniasis (PKDL) cases infected sandflies [23, 24]. However, only 48 asymptomatic individuals were tested and the experiments are ongoing.

Several studies have shown that proximity to past or current VL cases is a risk factor for infection and disease [15, 25–32] (see S1 Table). In addition to higher incidence of VL in households with past/current VL cases than households without VL, higher prevalence of positivity and incidence of conversion on diagnostic tests such as the rK39 ELISA, DAT, and leishmanin skin test (LST) have been observed in households with past/current VL cases, suggesting that transmission intensity is greater near VL cases. Picado et al [27] observed that incident VL was also associated with the presence of seropositive individuals in the same household at the baseline survey in their study (odds ratio (OR) for VL vs remaining DAT-negative = 2.43, 95% CI 1.55–3.79, p < 0.001) and the presence of other seroconvertors in the same household (OR = 2.22, 95% CI 1.38–3.58, p = 0.001) and within 50m of the household (OR = 8.73, 95% CI 2.59–29.41, p < 0.001) over the 2.5 years of the study. Incident asymptomatic DAT seroconversion was associated with presence of VL cases (OR = 1.66, 95% CI 1.16–2.36, p = 0.005), DAT-positive individuals (OR = 1.37, 95% CI 1.12–1.67, p = 0.002) and DAT seroconvertors (OR = 2.22, 95% CI 1.79–2.75, p < 0.001) in the same household. Incidence of asymptomatic infection and disease were more strongly associated with recent VL (i.e. VL that occurred in the relatively short time frame of the study) than VL that occurred in the 18 months before the study. These findings suggest that infection and transmission are highly spatially and temporally clustered.

Bern et al [15, 25] analysed spatial variation in risk of VL and asymptomatic infection in more detail using the dataset from Mymensingh, Bangladesh, analysed in this study. Associations between VL risk and asymptomatic infection risk (as measured by rK39 ELISA positivity or LST positivity) and distance from a VL case were assessed in terms of the odds ratios of having VL or being rK39 ELISA/LST-positive if living in the same household as, and a different household but <50m from, the closest previous VL case compared to living >50m from them. The risk of VL was substantially higher for individuals living in the same household as a previous case (OR = 6.37, 95% CI 3.30–12.28, p < 0.0001) and decreased relatively quickly with distance (OR = 1.85, 95% CI 0.95–3.60, p = 0.07, for individuals in a household without VL less than 50m from a case), while rK39 ELISA positivity and LST positivity were also strongly associated with proximity to previous VL cases. A multivariable logistic regression model comparing VL to recent asymptomatic infection (defined by rK39 ELISA positivity and LST negativity at baseline) showed that living in the same household as a previous case was associated with significantly increased risk of symptomatic infection (OR = 2.85, 95% CI 1.45–5.61, p = 0.003). Other studies have suggested that the risk of progression from infection to VL is higher for individuals living in households with past/current VL cases [28, 33, 34], and a study comparing transmission in households with recently treated VL cases against households with rK39-rapid-test-positive individuals or PKDL cases (or neither) found higher transmission (in terms of rK39-positivity) in VL households [35]. These studies lend support to the view that VL cases are the primary drivers of transmission, at least in highly endemic villages.

As VL incidence declines in the ISC, understanding spatial heterogeneity in transmission is particularly important for optimising control interventions. The main interventions currently employed are early case detection and treatment, and indoor residual spraying of insecticide (IRS) to try to reduce the density of the *Phlebotomus argentipes* sandfly vectors [6]. In endemic situations, the WHO recommendation is for IRS to be applied in affected villages and timed to precede maximum sandfly density, while during epidemics large-scale IRS covering all buildings, including houses and animal shelters is recommended [36]. An important question currently facing the control programme is whether more effort should be invested in improving case detection or IRS to reach and sustain the elimination target [7, 12, 20, 37]. This depends on the effectiveness of IRS, but also on the range of transmission around infected individuals and how much asymptomatic individuals contribute to transmission.

Spatially-targeted active case detection and IRS strategies are being considered as part of the national elimination programmes in India and Bangladesh due to resource constraints (the economic and practical infeasibility of performing active case detection and blanket spraying insecticide in all villages) and falling case numbers [3, 6, 9, 38, 39]. These strategies involve checking for infection and VL, and spraying insecticide, in all houses within a certain radius of a new VL case to try to limit transmission. Spatially targeted active case detection strategies have been tested in Bangladesh, India and Nepal with varying levels of success, potentially due to protocol differences (e.g. differences in the definition of ‘index’ cases, from any previous cases to only active cases, and the radii around index cases in which case detection was performed, from 50m to 200m), different levels of incidence, and implementation issues [40–42]. Currently, spatial targeting of IRS is being trialled only at village level in the Vaishali and Saran districts of Bihar state, India, with adjusted IRS village micro-plans in which all villages neighbouring (less than 500m from) a village with cases in the previous year are sprayed (based on an estimated maximum sandfly flight range of 500m [43–45]), along with all villages that have had any cases in the previous 3 years (as in the previous micro-plans) [38]. More precise estimates of the spatial and temporal range of transmission via sandfly dispersal are required to target IRS at a household level.

Although the epidemiological studies described above provide evidence of the importance of case proximity in transmission, they all use relatively simple statistical analyses with crude measures of spatial and temporal proximity to assess transmission patterns. In order to accurately account for variation in the infectious pressure on individuals in both space and time, a more fine-grained spatiotemporal model is required. Existing transmission models of VL [12, 18–20, 46, 47] fail to account for spatial heterogeneity in transmission, and as a result may overestimate the contribution of asymptomatic individuals to transmission [48]. Two key questions that need to be addressed are:

1. how does VL risk decrease with distance from a case?
2. how much do asymptomatic individuals contribute to transmission?

One approach that has been taken to estimate spatial variation in disease risk is using spatial kernel transmission models [49], in which the transmission rate between individuals is scaled by a function of the distance between them (the spatial kernel). Methods for inferring spatial kernels from geo-located incidence data were first developed for foot-and-mouth disease in livestock [50, 51], and have since been extended to handle a number of complexities, including missing data and unobserved infections [52–55], and been applied to other livestock diseases such as avian influenza and swine flu [56–58]. However, they have rarely been applied to human diseases [59] or vector-borne diseases [60] due to a number of challenges, including limited information on human movement [49]. Inference of spatial transmission of VL is particularly challenging due to long infectious periods, the long and highly variable incubation period (reported to last anywhere between 10 days and 2 years, though generally thought to be 2-6 months [25, 61], with an estimated average duration of 5 months (95% CI 4.3–5.5 months) based on diagnostic data [62]), the potential role of asymptomatic individuals in transmission, and the sparsity of data on the flight range of *P. argentipes* sandflies.

To start to address the questions above we have developed an individual-level spatial kernel transmission model for VL and fitted it to a detailed epidemiological dataset from a highly endemic setting in Bangladesh [15, 25] using a Bayesian inference framework. Our aim in developing and parameterising the model is to eventually use it to predict the impact of spatially-targeted control interventions in the ISC, once it has been validated against data from different settings.

## Methods

### Data

**Fig 1:**
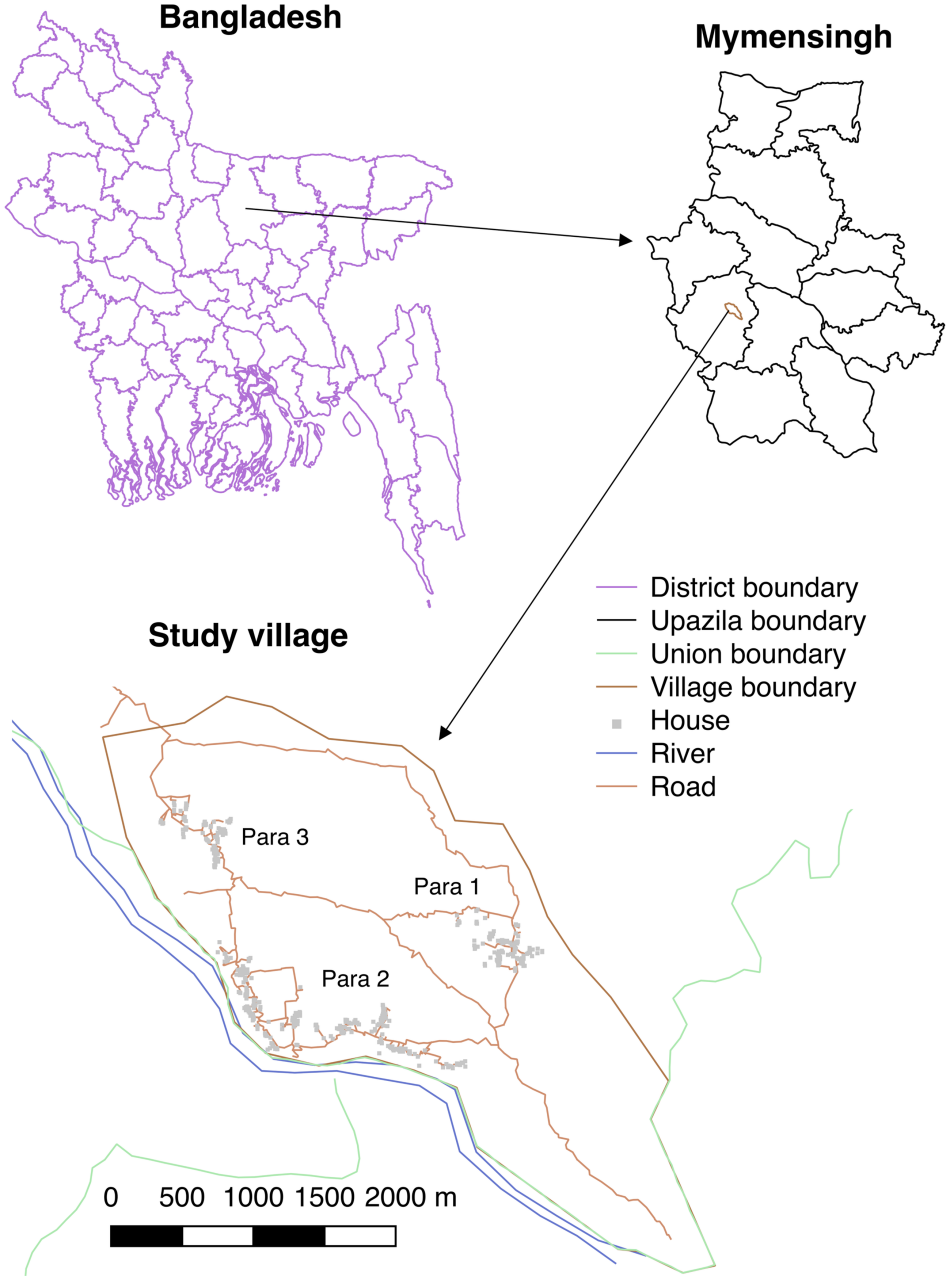
Location of study village and surveyed households in Fulbaria upazila, Mymensingh district, Bangladesh. District and upazila shape files from www.gadm.org/country, village details from GPS mapping during study. Maps produced using QGIS version 2.14.2 [64].

The data used in this study were collected in a longitudinal epidemiological study in a highly endemic village in Fulbaria upazila, Mymensingh district, Bangladesh between January 2002 and June 2004. Full details of the study design and data collection have been provided previously [15, 25, 62], but aspects particularly relevant to the present study are briefly described here. The data consist of detailed information on 2507 individuals living in 509 households in the 3 hamlets (or ‘paras’), out of the 9 in the village, that had the highest reported VL incidence in the years before the study. The 3 study paras are situated in an area approximately 2.7km×2km in size. Longitudinal VL incidence data—including dates of onset of symptoms, treatment, relapse, relapse treatment and death due to VL—was collected for 183 VL cases with symptom onset between January 1999 and June 2004 (retrospectively for those with onset before 2002), and onset years were recorded for 41 individuals who had VL prior to 1999 (see S1 and S3 Data). Annual censuses to record demographic information (births and deaths) and cross-sectional diagnostic surveys using rK39 ELISA and LST were conducted on the entire study population from January-April in 2002-2004 (S1 Data). All households present in 2002 were mapped with a Global Positioning System (GPS) device accurate to ±10m, and the GPS positions of households built in the study area after 2002 were imputed as being halfway between those of the two closest households (when these were known). Thus GPS coordinates were available for 506 of the 509 households (Fig 1). All analyses were restricted to the 2494 inhabitants of these households. Individuals were assigned GPS coordinates according to the household they belonged to and pairwise distances between all individuals calculated using the haversine formula [63].

### Model

**Fig 2:**
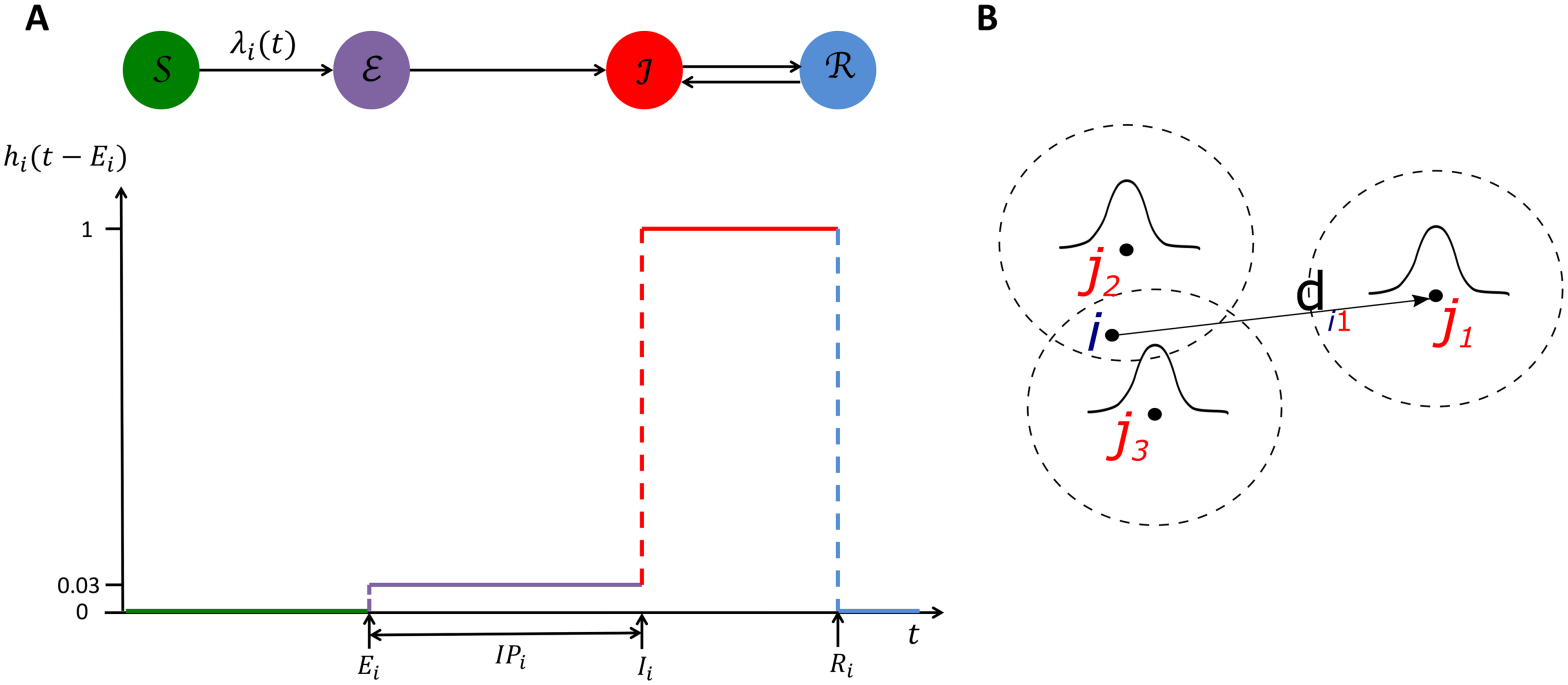
Schematic of the model structure. A: The different infection states in the model (susceptible S, pre-symptomatically infected E, symptomatically infected I, treated R) and their relative infectiousness *h*_*i*_(*t* − *E*_*i*_) over time *t* for a VL case *i* with infection time *E*_*i*_, incubation period *IP*_*i*_, symptom onset time *I*_*i*_ and treatment time *R*_*i*_. B: The infectious pressure λ_*i*_(*t*) on susceptible individual *i* at time *t* is the sum of the transmission rates from the infectious individuals around them (here *j*_*i*_, *j*_2_ and *j*_3_), which are a function (represented by the bell-shaped curves) of the distances of the infectious individuals from *i* (the arrow shows the distance *d*_*i*1_ between *i* and *j*_1_) and the times since their infections.

We describe VL transmission using an individual-level spatiotemporal susceptible-exposed-infectious-recovered (SEIR) model. In the model, individuals can be in one of four states at any particular time:

- susceptible (i.e. uninfected and without any history of VL, and able to become infected),
- exposed or pre-symptomatic (infected with the parasite and potentially infectious but without any symptoms),
- symptomatic (i.e. with clinical symptoms of VL and infectious),
- recovered (i.e. treated for VL or recovered from asymptomatic infection);

and can only progress from being susceptible to pre-symptomatically infected to symptomatic to recovered, and back to symptomatic if they suffer a relapse (Fig 2). Asymptomatically infected individuals are not explicitly modelled (see below for how transmission due to asymptomatic infection is implicitly incorporated). Note also that recovered individuals who do not relapse are assumed to possess lifelong immunity to VL. This is different to several previous transmission models of VL [18–20], which assume that individuals who have recovered from symptomatic or asymptomatic infection return to being susceptible after a number of years, and thus can be reinfected. Whilst this may be true for asymptomatic infection, multiple VL episodes are relatively rare [25] and the limited available evidence suggests that the majority of these are due to relapse rather than reinfection [65, 66]. PKDL cases are also not included, since there were only 4 confirmed cases of PKDL during the study period and no information was recorded on time of PKDL onset.

Time is measured in units of months (running from *t* = 1 (January 1998) to *t* = *T* = 78 (June 2004)) since this is a natural time scale for VL progression (the durations of infection and disease typically lasting for months) and is the finest time resolution to which some dates (such as birth and death) were recorded. We label individuals that developed VL during the study by *i* = 1, 2, …, *n*_*I*_, and the remainder of the study population by *i* = *n*_*I*_ + 1, *n*_*I*_ + 2, …, *n*, where *n* = 2494 is the total study population. The vectors of birth, infection, onset, treatment, relapse, relapse treatment, and death times of all individuals are denoted by **B** = (*B*_*i*_)_*i*=1, …, *n*_, **E** = (*E*_*i*_)_*i*=1, …, *n*_, **I** = (*I*_*i*_)_*i*=1, …, *n*_, **R** = (*R*_*i*_)_*i*=1, …, *n*_, $I_{R}={\left(I_{i}^{R}\right)}_{i=1,\ldots ,n}$, $R_{R}={\left(R_{i}^{R}\right)}_{i=1,\ldots ,n}$, and **D** = (*D*_*i*_)_*i*=1, …, *n*_. We define the sets of susceptible, pre-symptomatic, symptomatic and recovered individuals at time *t* by

$$\begin{array}{c} S(t)≔\{i:B_{i}\leq t<\text{min}\left(E_{i},D_{i}\right)\},\\ E(t)≔\{i:E_{i}\leq t<I_{i}\},\\ I(t)≔\{i:I_{i}\leq t<\text{min}\left(R_{i},D_{i}\right)\;\text{or}\;I_{i}^{R}\leq t<R_{i}^{R}\},\;\text{and}\\ R(t)≔\{i:R_{i}\leq t<\text{min}\left(D_{i},I_{i}^{R}\right)\;\text{or}\;R_{i}^{R}\leq t<D_{i}\},\;i=1,\ldots ,n, \end{array}$$

respectively, where we adopt the notation $E_{i}=I_{i}=R_{i}=I_{i}^{R}=R_{i}^{R}=\infty$ if individual *i* did not have VL during the study.

#### Transmission rates

In the model, susceptible individuals can become infected either from pre-symptomatic individuals (in E) (if they are assumed to be infectious), from symptomatic individuals (in I), or from ‘background’ transmission due to non-explicitly modelled factors, such as asymptomatic infection, human migration, and animal reservoirs (if any exist). It is commonly believed that transmission is anthroponotic in the ISC, i.e. that humans are the only reservoir of infection [4, 6]. Some studies have found animals positive for *L. donovani* DNA or antibodies against the parasite [67–70], but none have demonstrated that these animals are capable of infecting sandflies or that they play any role in transmission. The time-dependent transmission rate λ_*ij*_(*t*) between an infected individual *j* and a susceptible individual *i* is modelled as

$$\begin{array}{c} \lambda_{ij}\left(t\right)=\left(\beta K\left(d_{ij}\right)+\delta 1_{ij}\right)h_{j}\left(t-E_{j}\right),\;i\in S\left(t\right),\;j\in E\left(t\right)\cup I\left(t\right), \end{array}$$ (1)

where *β* is the overall rate constant for spatial transmission between infected and susceptible individuals; *K*(*d*_*ij*_) is the spatial kernel function that scales the transmission rate by the distance *d*_*ij*_ between individuals *i* and *j* (S2 Data); *δ* (≥ 0) is a rate constant for additional within-household transmission; **1**_*ij*_ is an indicator function for individuals living in the same household, i.e.

$$1_{ij}=\{\begin{array}{cc} 1, & \text{if}\;i\;\text{and}\;j\;\text{are}\;\text{in}\;\text{the}\;\text{same}\;\text{household},\\ 0, & \text{otherwise}; \end{array}$$

and *h*_*j*_(*t* − *E*_*j*_) is the infectiousness of the infected individual at time *t*. Thus, transmission from person to person through sandflies and spatial variation in this transmission due to sandfly movement is not modelled explicitly, but incorporated implicitly through the spatial kernel function. We note that this description relies on the assumption that humans are generally in fixed positions relative to each other when transmission to and from sandflies occurs. We assume this to be the case as the majority of sandfly biting occurs at night, typically between the hours of 9pm and 3am [71–76], when individuals are generally asleep inside or just outside their homes.

#### Spatial kernel

Given the limited flight range of the sandfly and evidence from previous studies that VL risk is high close to past/active cases and lower further away from them, it is reasonable to suppose that VL risk decreases with increasing distance from a case. We test this hypothesis by fitting the following different functions for the spatial kernel *K*:

- a constant kernel
  $$\begin{array}{c} K_{1}\left(d_{ij}\right)=\{\begin{array}{cc} 1, & \text{if}\;i\;\text{and}\;j\;\text{in}\;\text{same}\;\text{para},\\ 0, & \text{if}\;i\;\text{and}\;j\;\text{in}\;\text{different}\;\text{paras}, \end{array} \end{array}$$ (2)
- a Cauchy-type kernel
  $$\begin{array}{c} K_{2}\left(d_{ij}\right)=\{\begin{array}{cc} \frac{K_{0}}{1+{\left(d_{ij}/\alpha \right)}^{2}}, & \text{if}\;i\;\text{and}\;j\;\text{in}\;\text{same}\;\text{para},\\ 0, & \text{if}\;i\;\text{and}\;j\;\text{in}\;\text{different}\;\text{paras}, \end{array} \end{array}$$ (3)
- an exponential kernel
  $$\begin{array}{c} K_{3}\left(d_{ij}\right)=\{\begin{array}{cc} K_{0}\;\text{exp}\left(-d_{ij}/\alpha \right), & \text{if}\;i\;\text{and}\;j\;\text{in}\;\text{same}\;\text{para},\\ 0, & \text{if}\;i\;\text{and}\;j\;\text{in}\;\text{different}\;\text{paras}, \end{array} \end{array}$$ (4)
  where *α* is the parameter which determines the rate at which transmission risk decreases with distance (smaller values corresponding to a more rapid decrease); and *K*_0_, defined such that
  $$\sum_{i=1}^{n}\sum_{j=1,j\neq i}^{n}K\left(d_{ij}\right)=n,$$
  is a normalisation constant introduced to reduce correlation between *α* and *β* that negatively affects the mixing of the Markov chain Monte Carlo (MCMC) algorithm used to estimate the parameters (see Parameter Estimation). We choose the Cauchy-type and exponential kernels as they are commonly used kernels for modelling disease spread [53, 54, 58, 59, 77], and are monotonically decreasing functions of distance parameterised by a single parameter, so do not lead to parameter identifiability issues. We assume that (in the absence of human migration) cases can only infect other individuals in the same para based on the reported maximum flight range of *P. argentipes* sandflies in the Indian subcontinent being a few hundred metres [78–81], and the minimum distance between houses in different paras being 580m. Note that potential long-range transmissions between cases in different paras that might be missed by restricting the kernel in this way are covered by the inclusion of background transmission in the model (see below).

To test the null hypothesis that there is no spatial or temporal variation in transmission associated with VL cases, we compare the fit of the spatial kernel models defined above against a model with background transmission only (i.e. with *β* = 0 and *ϵ* > 0). The constant spatial kernel corresponds to the transmission rate in each para depending only on the number of pre-symptomatic and symptomatic individuals in the para, rather than on the positions of these individuals. Thus, testing the constant kernel against the Cauchy and exponential kernels enables an assessment of the evidence for individual-level spatial variation in transmission risk, as opposed to only para-level variation. The Cauchy kernel is a bell-shaped kernel, for which the risk is higher than the exponential kernel over short distances and also over long distances (due to its fatter tail), so we test both kernels to determine whether one provides a more accurate description of the variation in risk around a case. We also compare models with and without additional within-household transmission (*δ* > 0 and *δ* = 0) against each other to assess whether living in the same household as a case confers additional risk of disease, over and above that just due to distance from the case.

#### Infectiousness over time

The choice of the infectiousness function *h*_*j*_(*t* − *E*_*j*_) warrants discussion since very little is known about how infectiousness towards sandflies changes as infection progresses or how it varies between individuals for anthroponotic VL [82]. For canine VL, infectiousness is strongly associated with skin parasite load, which increases with the duration and severity of infection, and with clinical severity, but there is substantial variation between individual dogs [83]. In humans, the picture may be similar—disease severity generally increases with the duration of symptoms, though there is again substantial variation between individuals, and preliminary xenodiagnosis studies suggest that infectiousness is correlated with disease severity (as measured by parasite loads in splenic smears) [23]. For the infectiousness of pre-symptomatic individuals, only proxy measures, such as parasite loads in peripheral blood and antibody titres, are available. Parasite loads and antibody titres appear to be higher in pre-symptomatic individuals than asymptomatic individuals, but still several orders of magnitude lower than those in clinical cases [16, 84, 85]. Although modelling studies have not generally distinguished pre-symptomatic and asymptomatic infection, they suggest that pre-symptomatic individuals are much less infectious to sandflies than symptomatic individuals [18, 20, 48]. The infectiousness of VL cases who suffer relapse is also not known, but relapse appears to be associated with a resurgence in parasitaemia to high parasite loads [86, 87]. Whether these individuals are infectious between apparent clinical cure and recurrence of symptoms is unknown. Thus, in the absence of more detailed experimental data, we choose a simple step function for *h*_*j*_(*t* − *E*_*j*_) of the form

$$\begin{array}{c} h_{j}\left(t-E_{j}\right)=\{\begin{array}{cc} h_{0}, & \text{if}\;E_{j}\leq t<I_{j},\\ 1, & \text{if}\;I_{j}\leq t<R_{j}\;\text{or}\;I_{j}^{R}\leq t<R_{j}^{R},\\ 0, & \text{otherwise} \end{array} \end{array}$$ (5)

where *h*_0_ is the relative infectiousness of pre-symptomatic individuals compared to symptomatic individuals, cases are assumed to become uninfectious in their treatment month *R*_*j*_ (i.e. shortly after commencing treatment, based on observed rapid decreases in parasite loads with treatment [86, 88]), and we assume that relapse cases are as infectious upon relapse as in their first clinical episode, but uninfectious between treatment and relapse. Based on modelling estimates for the relative infectiousness of pre-symptomatic/asymptomatic individuals [19, 20] we choose *h*_0_ = 0.03, but also perform a sensitivity analysis for *h*_0_ to assess how it affects the parameter estimates (see S1 Text).

#### Incubation periods

Since the infection times of cases were not observed and the incubation period of VL is variable, the incubation period durations of the cases, *IP*_*j*_ = *I*_*j*_ − *E*_*j*_ (*j* = 1, …, *n*_*I*_), are random variables. We model them as independent negative binomial random variables with fixed shape parameter *r* = 3 and ‘success’ probability parameter *p* (and support starting from 1 rather than 0, such that the minimum incubation period is 1 month), i.e. *IP*_*j*_ ∼ *NB*(*r*, *p*), where *p* determines the mean incubation period (since E[NB(r,p)]=r(1-p)/p+1) and is estimated along with the transmission parameters *β*, *α*, *ϵ* and *δ* (see Parameter Estimation and S1 Text for further details). We choose this form for the incubation period distribution as it implies that very short or very long incubation periods are unlikely, but there is a certain intermediate incubation period duration (the mode of the negative binomial distribution) that cases are likely to have. This is more realistic than a geometric incubation period distribution (with *r* = 1), which would imply that the most likely infection time is the month before symptom onset (since it always has a mode at 1).

#### Infectious pressure

The ‘infectious pressure’ on susceptible *i* at time *t* is given by the sum of the transmission rates from all infected individuals at time *t* (see Fig 2) plus a constant background transmission rate *ϵ*

$$\begin{array}{c} \lambda_{i}\left(t\right)=\sum_{j\in E\left(t\right)\cup I\left(t\right)}\left(\left(\beta \;K\left(d_{ij}\right)+\delta 1_{ij}\right)h_{j}\left(t-E_{j}\right)\right)+\epsilon , \end{array}$$ (6)

where the background transmission rate is included to account for potential transmission from asymptomatic individuals (and from other factors, such as migration, that are not explicitly modelled). The transmission process is thus described by a discrete-time approximation to an inhomogeneous Poisson process with rate $\lambda \left(t\right)=\sum_{i\in S(t)}\lambda_{i}\left(t\right)$, such that the probability of susceptible individual *i* being infected in any particular month *t* is:

$$\begin{array}{c} p_{i}\left(t\right)=1-e^{-\lambda_{i}\left(t\right)},\;i\in S\left(t\right). \end{array}$$ (7)

Using this expression, and individuals’ imputed infection times if they had VL (see Parameter Estimation), the probability of each individual remaining susceptible or being infected in each month *t* ∈ {1, …, *T*} can be calculated (as *q*_*i*_(*t*) = 1 − *p*_*i*_(*t*) or *p*_*i*_(*t*) (*i* = 1, …, *n*) respectively), these monthly probabilities (and the probability of the individual’s incubation period duration if they had VL) multiplied together to give the likelihood for each individual’s infection course, and the likelihoods for all individuals multiplied together to give the overall likelihood of the data given the model (see S1 Text for full details and the likelihood expression).

#### Leishmanin skin test

The above model does not take into account the fact that some individuals develop immunity following asymptomatic infection. The LST is a test for previous *L. donovani* exposure and LST positivity is believed to represent durable cell-mediated immunity against the parasite, which may last for several years or for life in the absence of immunosuppression [32, 89]. The LST was applied and read at each of the annual cross-sectional surveys from 2002-2004 as described previously [62, 90]. Many individuals without past or subsequent VL were LST-positive at the first survey in 2002, and others who were seropositive and LST-negative at the first survey later became LST-positive without developing VL. It seems likely that these individuals possessed protective immunity against VL, at least for the period of the study, given that only 2 of 667 individuals without previous VL who were LST-positive at one of the surveys subsequently developed VL compared to 43 out of 1163 without previous VL who were LST-negative (relative risk of subsequent VL for LST-positives vs LST-negatives from 2002 survey = 0.05, 95% CI 0.006–0.33, p = 0.02).

To assess the impact on the parameter estimates of treating LST-positive individuals as immune, we incorporated the LST status data from the cross-sectional surveys into the model and re-estimated the parameters. The LST data was incorporated using a simple catalytic model for the age-prevalence distribution of asymptomatic LST-positive individuals, in which it is assumed that a certain proportion of infected individuals never become LST-positive (either because they do not develop cell-mediated immunity or because of poor sensitivity of the test); that the probability of being LST-positive otherwise only depends on age and increases monotonically with age; and that individuals who become LST-positive remain immune to VL for life. This is clearly a considerable simplification, since some studies have shown loss of LST-positivity over time [90–92] and it is uncertain whether this reflects loss of immunity or poor test sensitivity or both, and LST prevalence also varies in space and time as the force of infection changes, e.g. LST positivity is strongly associated with proximity to past VL cases [15, 32]. However, our aim is just to gauge whether immunity among asymptomatic LST-positive individuals affects estimates of the transmission rates and spatial kernel, rather than to provide an accurate model for LST conversion. Furthermore, the assumption of lifelong immunity following LST conversion, as opposed to only temporary immunity, should not significantly affect the parameter estimates, as it is likely that immunity lasted much longer than the study even if it waned (see S1 Text). Further details of the catalytic model and incorporation of the LST data are provided in S1 Text.

### Parameter estimation

#### Bayesian inference framework

We developed a Bayesian inference framework for estimating the model parameters from the data based on inference techniques for partially observed stochastic epidemics originally developed by O’Neill and Roberts [93] and since extended by others [52–54, 77, 94–97]. Variations of this framework have been used to analyse the spatial transmission of diseases such as measles, foot-and-mouth disease, avian influenza and American foulbrood in honeybees [53, 54, 95, 98–100].

Although the times of symptom onset and treatment, **I** and **R**, are known for most of the VL cases in the study, for some cases only their year of onset is known, and the infection times **E** of all the cases are unknown. Due to the long and variable incubation period of VL, it is necessary to impute this missing data as part of the algorithm for estimating the transmission parameters. A Bayesian approach provides a natural framework in which to do this, since model parameters are viewed as random variables and the unknown infection times and missing onset and recovery times (which we denote by **I**′ and **R**′ respectively) can be treated as extra parameters to be estimated via data augmentation [77]. With the model as described above, we specify the likelihood L(θ;A)≔P(A|θ) of the augmented or complete data **A** = (**U**, **Y**), consisting of the observed data $Y=(\tilde{I},\tilde{R},I_{R},R_{R})$ (with the observed onset and treatment times $\tilde{I}$ and $\tilde{R}$) and the unobserved data **U** = (**E**, **I**′, **R**′), given the transmission parameters and the incubation period distribution parameter, ***θ*** = (*β*, *α*, *ϵ*, *δ*, *p*) (see S1 Text). Using Bayes’ rule, this is then combined with prior distributions for the model parameters, P(θ), representing any prior information we have about their values, to give the joint posterior distribution P(θ,U|Y) of the parameters and unobserved data given the observed data:

$$\begin{array}{c} P(\theta ,U|Y)\propto P(U,Y|\theta )P(\theta )=L(\theta ;A)P(\theta ). \end{array}$$ (8)

In theory we would like to perform inference for ***θ*** by calculating the posterior distribution for ***θ*** given the observed data

$$\begin{array}{c} P(\theta |Y)\propto P(Y|\theta )P(\theta ). \end{array}$$ (9)

However, this would require integrating the likelihood of the observed data P(Y|θ) over the set of all possible values of the unobserved data, **Ω** (which is finite),

$$\begin{array}{c} P\left(Y|\theta \right)=\sum_{U\in \Omega }P\left(Y,U|\theta \right), \end{array}$$ (10)

and it is not computationally feasible to calculate this sum due to the very high dimensionality of **Ω**. Instead, we use a MCMC data augmentation scheme to sample from the joint posterior distribution for (***θ***, **U**) in Eq (8) by iteratively sampling from the conditional distribution of the parameters given the observed and missing data, P(θ|U,Y), and the conditional distribution of the missing data given the parameters and the observed data, P(U|Y,θ). Full details of the MCMC algorithm are given in S1 Text.

#### Prior distributions

To perform Bayesian inference we need to specify the prior distributions for the parameters ***θ*** = (*β*, *α*, *ϵ*, *δ*, *p*) (Table 1). As there was little information available with which to construct priors for the transmission rate parameters *β*, *ϵ* and *δ*, which are non-negative, we chose weak exponential priors for all three. For the spatial kernel parameter *α* we chose an exponential prior with mean 50m, Exp(1/50), based on Bern et al’s analyses of the same dataset [15, 25]. Since the incubation period parameter *p* ∈ (0, 1] is a probability, we chose a beta distribution as a conjugate prior for *p*. The parameters of the beta distribution were chosen to match the mean of the prior distribution for the incubation period (NB(*r*, *p*)) with the estimated mean incubation period from our previous analysis of the diagnostic data from the same study (∼5 months) [62].

**Table 1:** Definitions and prior distributions of estimated parameters.

Parameter (units)	Definition	Prior distribution
*β* (mnth^−1^)	Rate constant for spatial transmission from VL cases	Exp(1)
*α* (m)	Inverse distance decay rate of spatial kernel	Exp(1/50)
*ϵ* (mnth^−1^)	Background transmission rate	Exp(1)
*δ* (mnth^−1^)	Additional within-household transmission rate	Exp(1)
*p*	Incubation period distribution parameter	Beta(22, 28)

### Model comparison

We compared the goodness of fit of the background-transmission-only model and the different spatial kernel models defined by Eqs (2)–(4), with and without additional within-household transmission, using the Deviance Information Criterion (DIC) [101]. DIC measures the trade-off between model fit and model complexity, and is the Bayesian equivalent of the Akaike Information Criterion (AIC) [102]. Since some data is unobserved, we required a modified version of the standard DIC that takes into account the missing data. We used a version of DIC for missing data models proposed by Celeux et al [103] that is based on the complete data likelihood *L*(***θ***; **A**) and calculated using the modes of the posterior distributions for the parameters (see S1 Text for further details). As for AIC, lower DIC values indicate better model performance, though differences of less than 5 units between models do not suggest a substantial difference in goodness-of-fit [104]. Given that there are potential issues with using DIC for model comparison [105], we also compared the posterior distributions of the deviances of the models to assess differences in quality of fit, where the deviance is defined up to an additive constant as

$$\begin{array}{c} D(\theta )=-2\;\text{log}\;(L(\theta ;A)). \end{array}$$ (11)

## Results

**Fig 3:**
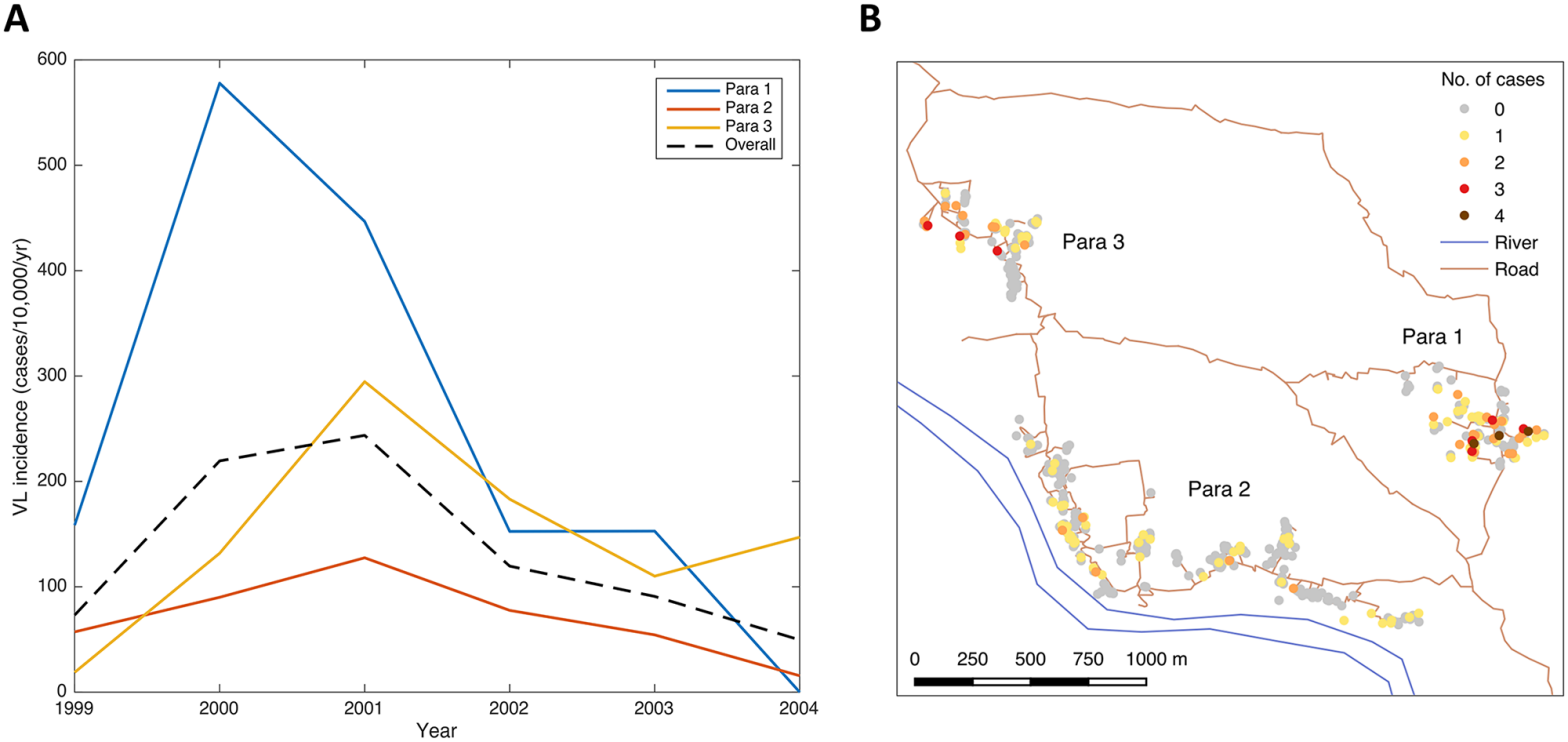
Para- and household-level VL incidence, January 1999—June 2004. A: Annual incidence in each para. B: Total number of VL cases in each household.

**Fig 4:**
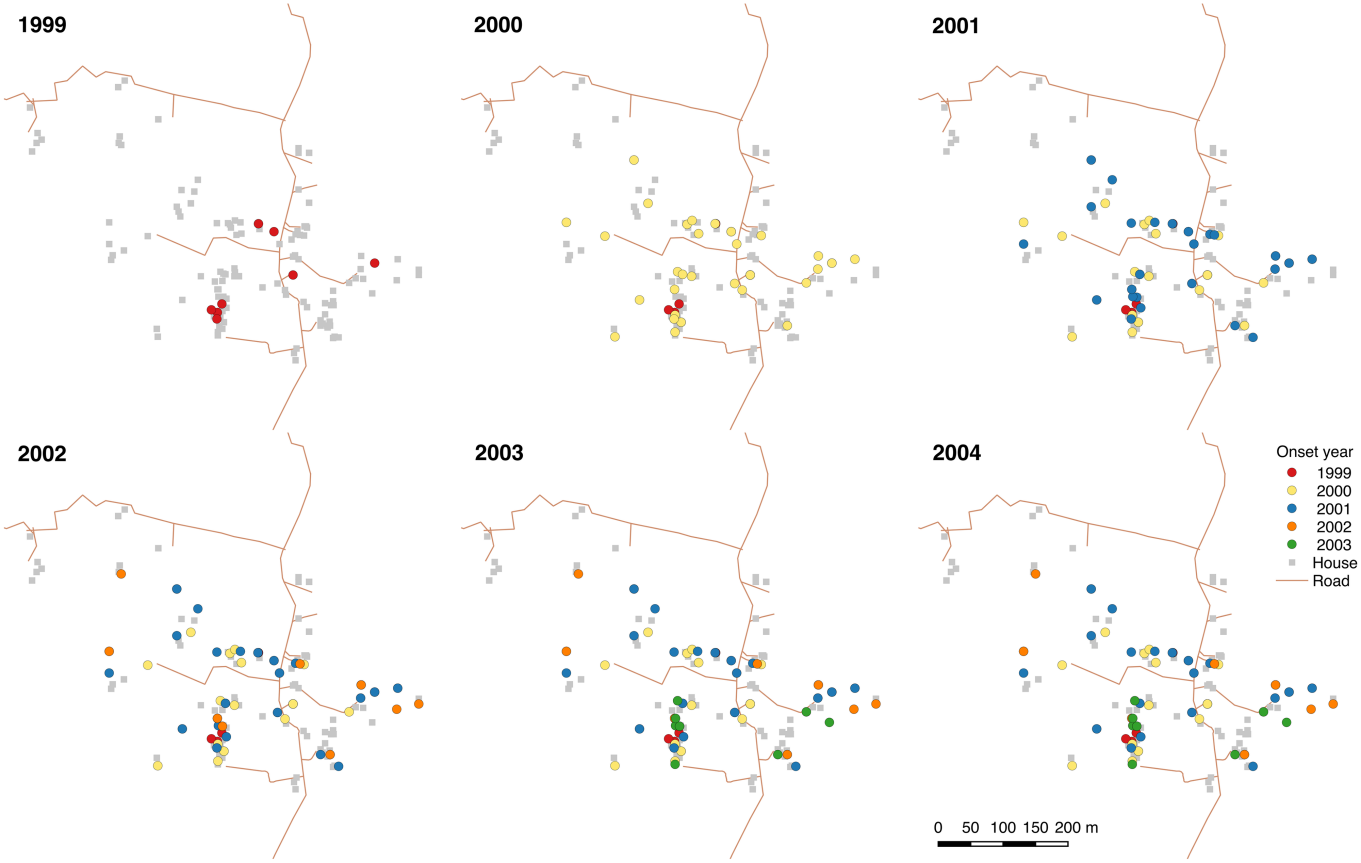
Locations of VL cases in para 1 by year of onset, 1999-2004. There were no VL cases with symptom onset in para 1 from January-June 2004. Maps for paras 2 and 3 are shown in S1 and S2 Figs. Locations of roads and households taken from GPS mapping during study.

**Fig 5:**
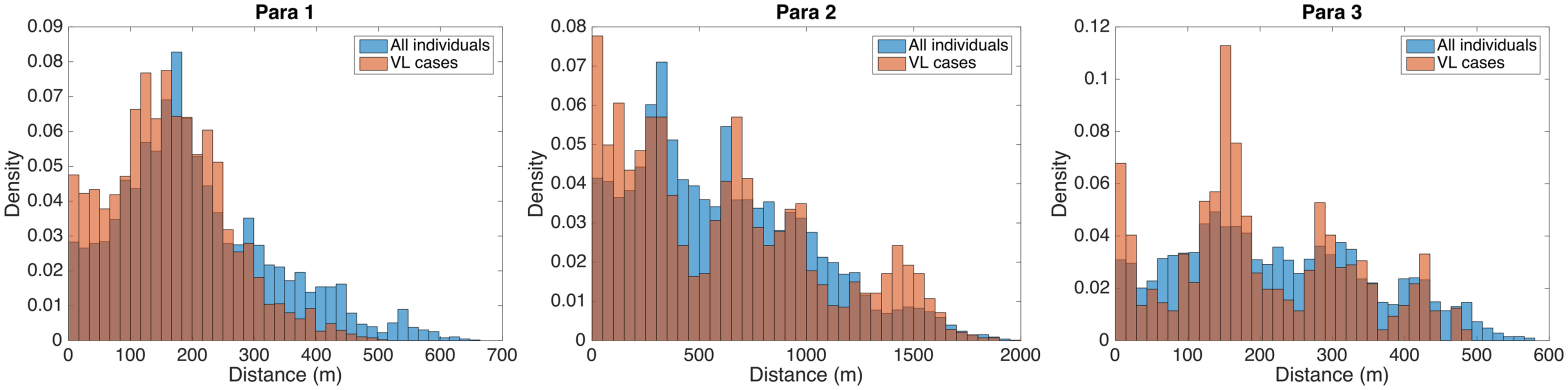
Distributions of pairwise distances between all individuals and between all VL cases in each para.

Demographic and VL incidence data for the three paras are presented in Table 2 and Fig 3. Average household size was relatively consistent across the three paras, but there were considerable differences in VL incidence and the average number of VL cases per household with VL. All three paras had high incidence from January 1999 to June 2004, but para 1 had the highest average incidence (248/10,000/yr) and average number of cases per VL household (1.65), and para 2, the largest para, the lowest (70/10,000/yr and 1.10 respectively). Most of the difference in incidence was due to much higher incidence in para 1 between 1999 and 2002, prior to the start of the study (Fig 3A). The para-level incidences are similar to those that were observed during 1-3 year ‘micro-epidemics’ in highly endemic clusters of hamlets in the data from Muzaffarpur, Bihar State, India, analysed by Bulstra et al [106]. Fourty-one households, 29 of which were in paras 1 and 3, had multiple cases between 1999 and 2004 (Fig 3B). Paras 1 and 3 also had greater maximum numbers of cases per household: all 3 households that had 4 cases were in para 1 in close proximity to each other and several households that had 3 and 2 cases, and there were 3 households that had 3 cases in para 3, but no household in para 2 had more than 2 cases. The evolution of the spatial pattern of VL cases in each para (Fig 4 and S1 and S2 Figs) from 1999-2004 suggests highly focal transmission around VL cases, with new cases in each year generally appearing very close to VL cases with onset in the previous year. Fig 5 shows the distributions of the distances between all pairs of individuals and all pairs of VL cases for each of the paras. The differences between the distributions for each para, e.g. the higher density in the case distribution at short distances (<100m) and the clumping of the case distribution, reflect the high degree of spatial clustering of cases.

**Table 2:** Demographic and VL incidence data for the three study paras for January 1999—June 2004.

Para	No. of HH (%)	Population in 2002	No. of VL cases	No. of VL HHs (%)	No. of cases per VL HH	Mean VL incidence (cases/10,000/yr)
1	126 (24.9)	590	86	52 (40.3)	1.65	248
2	272 (53.8)	1289	53	48 (37.2)	1.10	70.4
3	108 (21.3)	546	44	29 (22.5)	1.52	135
Total	506 (100)	2425	183	129 (100)	1.42	129

HH = household, VL HH = household with ≥ 1 VL case.

### Parameter estimates

**Fig 6:**
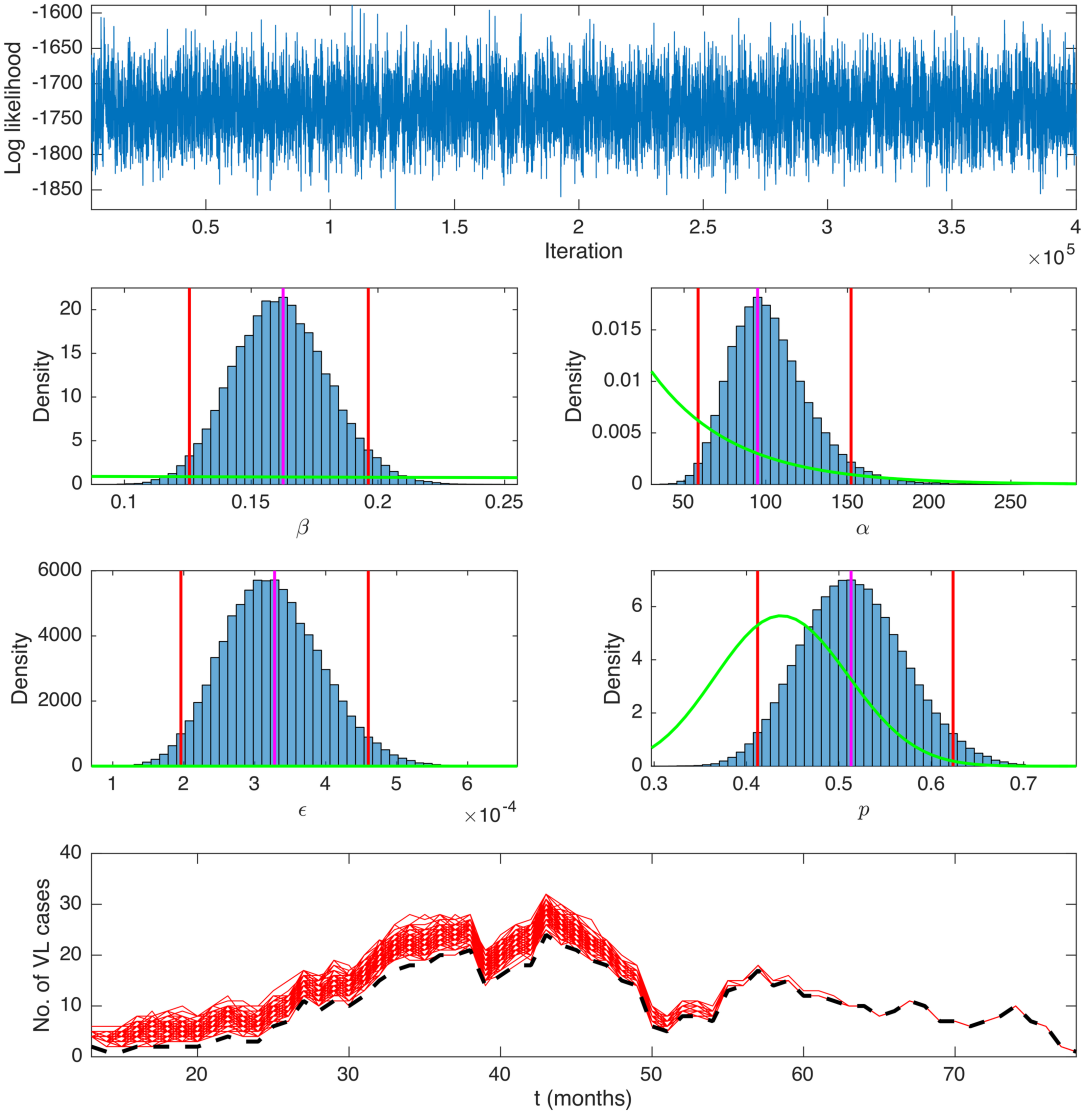
Output of the MCMC algorithm for the exponential kernel model. Top: Log-likelihood trace plot. 2nd-4th row: Posterior distributions for the spatial transmission rate constant *β* (mnth^−1^), risk decay distance *α* (m), background transmission rate *ϵ* (mnth^−1^), and incubation period distribution parameter *p*, with prior distributions (green lines), posterior modes (magenta lines) and 95% highest posterior density intervals (red lines). Bottom: Number of active VL cases over time in 100 iterations of the MCMC algorithm (red lines), with imputed missing onset and recovery times, and number of active cases excluding individuals with missing onset and/or recovery times (black dashed line).

Fig 6 shows the output of the MCMC algorithm for the exponential kernel model, including the log-likelihood trace plot and the posterior distributions for the transmission parameters, *β*, *α*, and *ϵ*, and incubation period distribution parameter, *p*. It is clear from the plots that the MCMC chain converged rapidly and mixed well, and that the parameters are well defined by the data. The chain was initialised from various regions of parameter space to assess convergence and in all cases nearly identical distributions for the parameters and log-likelihood were obtained.

The estimates (posterior modes and 95% highest posterior density intervals (HPDIs)) for the transmission parameters and DIC for each of the models tested are presented in Table 3. Comparing the DIC values for the different models, it is clear that those in which transmission risk decreases with distance from a VL case fit the data much better than the models with no variation with distance (i.e. the ‘background-only’ model and constant kernel model, for which the differences in DIC from the best-fitting model are ΔDIC = 247.1 and ΔDIC = 64.1 respectively). The best-fitting model is the exponential kernel model. However, the DIC differences between this model and the Cauchy kernel model and the models with additional within-household transmission are all small (ΔDIC < 7), and their deviance distributions are virtually completely overlapping (S3 Fig), suggesting that the data is similarly well described by a Cauchy or exponential kernel and models with additional within-household transmission.

**Table 3:** Parameter estimates for different models fitted to the data.

Model	Parameter estimates*								DIC
	*β* (mnth^−1^)	95% HPDI	*α* (m)	95% HPDI	*ϵ* (×10^−4^ mnth^−1^)	95% HPDI	*δ* (×10^−3^ mnth^−1^)	95% HPDI	
Background-only	0^†^	-	-	-	11	(9.2–12)	0^†^	-	3706.6
Constant kernel	0.24	(0.18–0.29)	-	-	2.4	(1.1–4.0)	0^†^	-	3523.6
Decreasing VL risk with distance									
Cauchy kernel	0.17	(0.13–0.21)	64	(38–134)	3.0	(1.8–4.5)	0^†^	-	3466.5
Exponential kernel	0.16	(0.13–0.20)	95	(59–152)	3.3	(2.0–4.6)	0^†^	-	3459.5
Additional within-household transmission									
Cauchy kernel	0.16	(0.12–0.20)	80	(40–148)	3.1	(1.8–4.4)	1.6	(0.1–4.5)	3462.7
Exponential kernel	0.15	(0.12–0.19)	102	(62–160)	3.1	(1.9–4.5)	1.4	(0.1–4.4)	3459.7
LST status data included									
Cauchy kernel	0.24	(0.19–0.30)	81	(44–139)	3.5	(2.0–5.4)	0^†^	-	-^‡^
Exponential kernel	0.23	(0.18–0.28)	100	(69–157)	3.8	(2.4–5.7)	0^†^	-	-^‡^

* Modes of marginal posterior distributions for parameters and 95% highest posterior density intervals (HPDIs).

^†^ Assumed.

^‡^ No DIC calculated as additional data included in the fitting.

The parameter estimates are highly consistent across the Cauchy and exponential kernel models. The estimates for the spatial transmission rate constant *β*, background transmission rate *ϵ* and additional within-household transmission rate can be interpreted as follows. In the absence of any background transmission, the estimated monthly probability of developing VL from living in the same household as a single symptomatic case was 14–16 in 10,000 (95% HPDI 6–31 in 10,000) according to the models without extra within-household transmission, and 26–28 in 10,000 (95% HPDI 7–70 in 10,000) according to models with extra within-household transmission. The monthly probability of developing VL from background transmission if there were no VL cases nearby was approximately 3 in 10,000 (95% HPDI 1.8–4.6 in 10,000). Put another way, the models suggest that at least 134–138 of the 183 VL cases in the 3 paras from 1999-2004 were infected due to being near to VL cases, while the remaining cases originated from background transmission (or at least cannot be explained by spatial proximity to active VL cases or infectious pre-symptomatic individuals).

**Fig 7:**
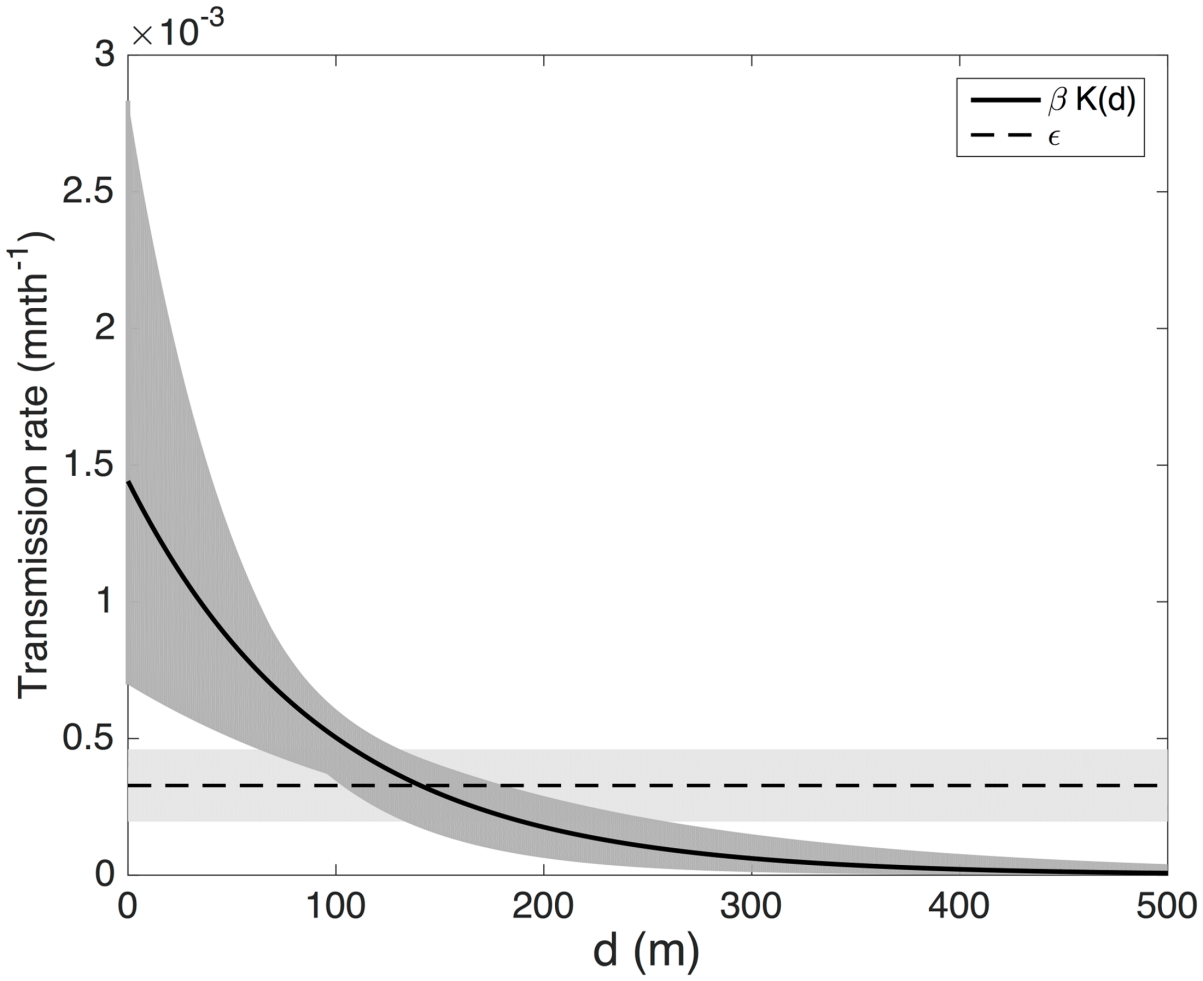
Estimated spatial transmission kernel for the exponential kernel model. Kernel (solid black line) shown as the transmission rate per month, *βK*(*d*), as a function of distance *d* from a single VL case. Modal estimate for kernel and 95% highest posterior density interval (HPDI) (dark grey shaded region) calculated from posterior modes and HPDIs for the transmission parameters. The mode (dashed black line) and 95% HPDI (light grey shaded region) for the background transmission rate, *ϵ*, are also shown for comparison.

All but one of the posterior modes for the distance decay rate parameter, *α*, are ≤ 100m, suggesting VL risk decreased relatively quickly with distance from a case. Although the *α* estimates for the exponential kernel models are greater than those for the Cauchy kernel models, they actually correspond to a very similar rate of decrease in risk, due to the different shapes of the kernels. Accounting for background transmission, the estimates for the distance at which the risk of VL halves compared to living in the same household as a case (the ‘half-risk distance’) are 79m (95% HPDI 38–197m) and 87m (95% HPDI 50–166m) for the Cauchy and exponential kernel models without extra within-household transmission, and 33m (95% HPDI 4–105m) and 0m (i.e. double the risk in the same household as a case) (95% HPDI 0–69m) for the models with additional within-household transmission. Fig 7 shows the estimated spatial kernel (in terms of the transmission rate from a single VL case *βK*(*d*) a distance *d* away) and its 95% HPDI, for the exponential kernel model. The relatively rapid decay in risk with distance is clear, but there is considerable uncertainty in the estimated transmission rate up to 100m. The estimated background transmission rate and its 95% HPDI are also shown, indicating that the transmission rate from VL cases is higher than the background rate up to around 150m from a case (Box 1).

**Box 1. What are potential drivers for spatiotemporal clustering of VL at household level?**

- Recent cases (with symptom onset within the last year) in the same or nearby households (within 150m).
- Living in the same household as a case may lead to additional VL risk over and above that due just to distance from a case, but the magnitude of any risk increase is highly uncertain.
- Poverty? No significant differences in VL risk by income, education, occupation, land ownership or asset ownership were observed in single-variable and multivariable logistic regression models of the same data [15, 25], though more frequent beef or goat consumption was found to be associated with reduced risk of progression from infection to VL [15].

Including additional within-household transmission in the model led to an increase in the estimates for *α* since a flatter kernel shape was required to compensate for the extra within-household transmission. The estimate for the additional within-household transmission rate *δ* for the exponential kernel model suggests an approximately 2-fold increase in risk (95% HPDI 0.2–5.4) from occupying the same household as an active case compared to living right next to a case, but this result should be interpreted with caution given the wide 95% credible interval and the fact that this model did not fit significantly better than the models without additional within-household transmission (Box 1).

Including the LST data and treating individuals as immune upon becoming LST-positive did not significantly affect the estimates for *α* and *ϵ*, which both increased by only a small amount (Table 3). However, it resulted in an increase in the estimate for *β*, such that the estimated monthly probability of developing VL living in the same household as a case increased (from 14 in 10,000 to 19 in 10,000 for the exponential kernel model). This is as expected, given the reduction in the susceptible population from treating LST-positive individuals as immune and the proximity of a large proportion of the LST-positive individuals to VL cases [15].

The posterior distribution for the incubation period distribution parameter, *p*, was very similar for all models apart from the background-only model (posterior modes 0.50–0.51, 95% HPDIs (0.40–0.62)–(0.41–0.63)), suggesting that the incubation period distribution was relatively well constrained by the data given its assumed shape. The estimated posterior distribution for *p* corresponds to a mean incubation period duration of approximately 4 months (95% HPDI 2.7–5.1 months).

## Discussion

This study is the first individual-level fully spatiotemporal analysis of VL transmission in the Indian subcontinent and the first attempt to estimate a spatial transmission kernel for VL. It builds upon the work of several field studies that have identified proximity to a recent or current VL case as a strong risk factor for infection and disease, and given estimates of the increase in risk from living with or nearby a VL case, by providing a framework for a more refined spatiotemporal analysis of VL transmission. In particular the framework we have developed accounts for the large variation in the incubation period of the disease, the unobserved infection times of cases, the number of individuals infectious at the infection time of each new case and their distances from the new case, and potential transmission from asymptomatic individuals.

The results of our analysis suggest that the risk of being infected and developing VL decreases relatively quickly with distance from a case (halving within 90m) in a high endemicity setting. This estimate broadly agrees with the results of previous studies that used different methods of analysis. A multivariable logistic regression model of VL risk for the same dataset [15], found that the OR for VL, relative to living more than 50m from a previous case, decreased from 6.37 in the same household as a case to 1.85 in a household within 50m of a case. Another study on data from India and Nepal also found elevated risk of VL when living within 50m of a case with VL in the previous 18 months, compared to being more than 50m away (OR = 2.14, 95% CI 1.27–3.62, *p* = 0.004) [27]. Given that the spatial kernel in our transmission model represents the spread of infection from person to person due to the sandfly movement, the estimated kernel is also in agreement with the results of recent mark-release-recapture sandfly dispersal studies performed in Bihar, India, in which most sandflies only flew short distances (∼20m), and very few more than 150m [78]. Nevertheless, the estimated distance from a case over which risk decays is relatively small, and it is possible that other aspects of the local environment, such as housing density and configuration, are important in transmission at this scale. In future work we will investigate the potential role of these fine-scale environmental factors in the spatial patterns of VL cases.

The estimated mean incubation period of 4 months is consistent with our previous estimate of ∼5 months (95% CI 4.3–5.5 months) from a multi-state Markov model of the individual-level diagnostic data (including the serological data, which was not used in the present analysis) and case onset and treatment data from the same study [62], and falls within the 2-6 months range reported as typical in the literature [25, 61]. It also appears to agree relatively well with data from longitudinal studies on times from being measured sero-/PCR-positive to developing VL [26, 28, 33, 34, 107–109], which suggest most disease conversion occurs within 6 months.

The estimated relative contributions of transmission from nearby VL cases and background transmission to the observed number of cases suggest that VL cases contribute significantly more to transmission than asymptomatic individuals (at least three times as much) when incidence is high. Given that the background transmission rate also covers potential transmission from animals, the fact that it is much smaller than the case-to-case transmission rate suggests that the contribution of animals to transmission (if it exists) is small. Together with the relatively limited range of transmission around VL cases implied by the estimated spatial kernel, this suggests that reactive spatially-targeted interventions could be effective for reducing VL risk (Box 2). However, the relationship between reaction time to new cases and the radius around them in which interventions would need to be performed is uncertain and needs quantification [110]. It is likely that such a strategy would rely on early detection of new cases and interventions being performed soon after their detection and in a large radius around them (≥ 300m according to the estimated kernel). Even short delays could render the strategy ineffective, since by the time spraying and active case detection were performed the index cases would probably have already infected the next generation of cases within their transmission range.

**Box 2. What do spatiotemporal patterns at household level tell us about VL transmission dynamics?**

In a highly endemic setting:

- VL risk decreases relatively quickly with distance from a VL case (halving within 90m).
- VL cases appear to contribute more to transmission than asymptomatic individuals.
- Spatially-targeted interventions could be effective for reducing VL risk if performed promptly and in a sufficiently large radius around a new case.

With regard to implications for spatially-targeted control, it is important to note that the estimated kernel is a single static estimate of how VL risk varies with distance from a case based on incidence over 5.5 years in a highly endemic setting. This is also true of the estimated asymptomatic contribution. How spatial transmission patterns and the asymptomatic contribution vary across settings with different endemicities and longer periods of time is uncertain. The approach used here should be applied to more recent data and data from other geographical areas to assess this variation. This is particularly important given the large differences in incidence between Bangladesh, India and Nepal and the significant declines in incidence in all three countries since the study was conducted. Although existing data suggests that proximity to VL cases is a strong risk factor for infection in all three countries and across different endemicity levels [15, 26, 27, 111], it is possible that spatial patterns of transmission and the asymptomatic contribution have changed as incidence has declined, and further work is needed to assess the generalisability of the results to low-incidence and outbreak settings. Bern et al [25] observed that even over the course of the study, the pattern of VL cases spread from being highly-clustered to saturating major parts of each para (see Fig 4, S1 and S2 Figs), as a large proportion of the population became exposed and those that did not develop disease gained some level of immunity to the parasite. They suggested that this saturation could occur within 2-3 years in high transmission intensity settings, which agrees with observations of 1-3 year micro-epidemics in hamlets in Muzaffarpur, Bihar, India [106]. Thus, spatially-targeted interventions would need to be implemented as early as possible in a micro-epidemic to maximise their impact.

Another important consideration is the practical and economic feasibility of performing spatially-targeted IRS and case detection at a sub-village level, as it would require the ability to mount a rapid response to new cases and delineate the households that would receive the interventions, and focal active case detection could be time-consuming and expensive to implement. Given that VL incidence is also strongly clustered at hamlet level [106], it might make more sense to implement targeted control at this level depending on resource constraints. Based on our estimates, the policy of spraying all villages within 500m of villages with cases in the previous year that is currently being trialled in India seems sensible, as it should account for the fact that the disease may have been transmitted through more than one generation of cases by the time the spraying is performed.

Given the uncertainties in the potential impact of reactive focal IRS and active case detection and in the time window after detection of a new case in which they would be effective, and the difficulties of trialling them, in future work we will predict the impact of different spatially-targeted control strategies by simulating them with the parameterised spatiotemporal transmission model.

### Limitations of this analysis

As with any spatiotemporal analysis of transmission of a disease such as VL, with a complicated natural history and transmission cycle, our analysis has a number of limitations. In particular, we have not included the serological data from the annual cross-sectional surveys in the model and have not explicitly modelled asymptomatic infection, but incorporated potential transmission from asymptomatic individuals via a background transmission rate that is constant in space and time. Although we have considered the implications of LST-convertors being immune, this was via a very simple age-dependent model for the probability of being LST-positive, which does not take account of spatial and temporal heterogeneity in the force of infection. Asymptomatic infection is known to be associated with spatial and temporal proximity to VL cases, so if asymptomatic individuals transmit it is likely that their contribution varies in space and time. Incorporating the diagnostic data into the model would enable explicit modelling of asymptomatic infection and could help to constrain the possible infection times of cases (e.g. by assuming that individuals cannot be seropositive before they are infected). However, constructing the model and performing the inference on the full data is technically very challenging since the diagnostic surveys only cover the prospective part of the study (2002-2004), the tests (in particular the LST) have imperfect sensitivity and specificity, many individuals are missing tests, and there are a large number of observed combinations of test results. An inference approach that can account for unobserved infections, such as reversible jump MCMC [53, 54], would thus be required, along with a hidden Markov model for the true infection status underlying the observed test results [97]. Both the infection and recovery times of asymptomatically infected individuals are unobserved, so it would also be necessary to impute these from the diagnostic data.

Potential edge effects in space—contributions from infected individuals in unsurveyed paras neighbouring the study paras—are not accounted for in the model. Based on evidence that *P. argentipes* flies have a limited flight range and the study paras being more than 500m apart, we assumed that there was no transmission between cases in different paras in the absence of migration. However, some of the unsurveyed paras are within the maximum flight range of the sandfly and had cases during the study period, so it is possible that there was inter-para transmission from infectious individuals in households near the edges of unsurveyed paras. A related issue is that the model does not explicitly account for migration (which is covered via the background transmission rate), and assumes individuals were infected in or very nearby their households. In reality, there is a significant amount of seasonal migration for work in rural Bangladeshi communities, which may affect transmission, and there is uncertainty about how much transmission occurs indoors vs. outdoors [14]. These issues will be addressed in future work analysing spatial transmission over a longer period in the same paras and the unsurveyed paras using geo-located incidence and migration data [112].

Another limitation is that the model treats VL risk as purely a function of the local number of infectious individuals and their spatial proximity, and does not include other potential risk factors such as age, sex, bed net use and socioeconomic status. Such risk factors can be included in the model by treating the transmission rate between individuals (λ_*ij*_(*t*) in Eq (1)) as a function of these variables, but were omitted here to keep the analysis as simple as possible and because case proximity was identified as the strongest risk factor in the original analyses [15, 25]. Sandfly density, which varies seasonally [113], is also likely to affect transmission risk, but sandflies are not explicitly represented in the model due to a lack of comprehensive data on sandfly abundance in the study paras, and uncertainties in the relationship between sandfly and host densities and infection prevalences [12, 14] (Box 3).

**Box 3. What can we learn from this analysis for future data collection?**

- Further epidemiological studies to collect geo-located VL incidence data are required to assess how spatial clustering of VL varies with endemicity and over long periods of time.
- Measurements of sandfly densities and infection prevalence should be performed alongside measurements of human infection and disease, to assess the role sandfly bionomics play in spatial clustering.
- More detailed studies of the dispersal and flight range of *P. argentipes* sandflies are required to determine how far they can spread the parasite and over what timescale.

The unknown infectiousness profile of VL cases over time (before and during symptoms) and how it varies between individuals is also a potential source of uncertainty in the estimated spatial kernel and transmission rates. In households with multiple cases during the study period, there were long intervals between the onsets of the cases (longer than would be expected if the later cases were infected by the earlier cases, based on the estimated incubation period), possibly suggesting that some cases can remain infectious even after apparently successful treatment, although this is complicated by potential transmission from asymptomatic individuals and other nearby cases.

Finally, we note that the results of this analysis need to be validated. Unfortunately, there are relatively few geo-located VL incidence datasets available with which to do this. However, as part of a more formal validation of the model, in future work we will implement the equivalent stochastic spatial simulation model and test that it reproduces similar spatial patterns of incidence to those observed.

## Conclusion

Our analysis shows that in a high-endemicity setting VL transmission is focused around cases and cases are the main drivers of transmission. This suggests that reactive spatially-targeted control interventions could be effective at reducing transmission in high endemicity areas if implemented promptly and in a sufficiently large area around a case. Nevertheless, it is necessary to validate the model against data from different settings and to use it to predict the impact of different control strategies to assess whether spatially-targeted interventions would be effective, and under what conditions. If targeted interventions were predicted to be effective, the results could then be used to inform control policy to help achieve and maintain the 2020 elimination target for VL in the ISC.

## Supporting information

S1 Table Data on impact of case proximity on VL risk. Odds ratios for VL and asymptomatic infection risk based on proximity to infected individuals (from previous studies).
(XLSX)

S1 Text Supplementary information on model and parameter estimation. Definitions of model likelihood and deviance information criterion, and details of MCMC algorithm.
(PDF)

S1 Data Data used to fit model. Data on visceral leishmaniasis status and leishmanin skin test status of 2494 individuals in the study area, including dates of onset, diagnosis, treatment, and, where applicable, relapse and treatment for relapse for 183 VL cases.
(CSV)

S2 Data Matrix of pairwise distances between all individuals in the study. Distances are given in the same order as individuals are listed in S1 Data.
(ZIP)

S3 Data Metadata for S1 and S2 Data. (CSV)

S1 Fig Locations of VL cases in para 2 by year of onset, 1999-2004. (PDF)

S2 Fig Locations of VL cases in para 3 by year of onset, 1999-2004. (PDF)

S3 Fig Deviance distributions for the different models. (PDF)
