# Supplementary material for: The role of case proximity in transmission of visceral leishmaniasis in a highly endemic village in Bangladesh
Source: PLoS Negl Trop Dis. 2018 Oct 8;12(10):e0006453. doi: 10.1371/journal.pntd.0006453 (PMC6175508; doi:10.1371/journal.pntd.0006453)
Supplement: S1 Text — Definitions of model likelihood and deviance information criterion, and details of MCMC algorithm. (PDF) [file pntd.0006453.s002.pdf]

The role of case proximity in transmission of visceral leishmaniasis in a highly endemic village in Bangladesh: S1 Text

Lloyd A.C. Chapman, Chris P. Jewell, Simon E.F. Spencer, Lorenzo Pellis, Samik Datta, Rajib Chowdhury, Caryn Bern, Graham F. Medley, T. Déirdre Hollingsworth

Contents

1 Overview **2**

2 Further model details **2**
2.1 Incubation periods . . . 2
2.2 Missing onset and treatment times . . . 2
2.3 Catalytic model for LST data . . . 3
2.4 Complete data likelihood . . . 4

3 Parameter estimation **6**
3.1 Bayesian inference . . . 6
3.2 MCMC data augmentation scheme . . . 6
3.2.1 Initial parameter values and missing data values . . . 6
3.2.2 Prior distributions . . . 7
3.2.3 MCMC Algorithm . . . 7
3.2.4 Adaptive random walk Metropolis-Hastings . . . 9

4 Model comparison **9**
4.1 Deviance information criterion for missing data models . . . 9

5 Further details on results **10**
5.1 MCMC output . . . 10
5.1.1 Unobserved infection, onset and treatment times . . . 10
5.1.2 Parameter correlation . . . 11
5.2 Sensitivity analysis for pre-symptomatic infectiousness, h_0 . . . 12

1 Overview

This file provides further information on the spatiotemporal transmission model and Bayesian inference framework described in the Methods section in the main paper, including the expression for the likelihood of the model; details of the MCMC algorithm used to estimate the model parameters defined in Table 1 in the main text; further details on the use of the deviance information criterion for comparing the different models fitted to the data; and additional output from the MCMC scheme.

2 Further model details

2.1 Incubation periods

To account for the fact that the incubation period of VL is relatively long (typically several months) and highly variable, and infection times were unobserved, we model the incubation period $IP = I - E$ using a negative binomial distribution $\mathrm{NB}(r, p)$ with fixed shape parameter $r = 3$ and 'success' probability parameter p, i.e. via the probability mass function:

$$f(x) = \mathbb{P}(IP = x) = \frac{\Gamma(r + x - 1)}{(x-1)!\Gamma(r)} p^r (1-p)^{x-1}, \quad x \in \{1, 2, 3, \dots\} \tag{1}$$

Since there is uncertainty in the incubation period distribution, the parameter p is estimated as part of the MCMC algorithm for inferring the transmission parameters and missing data (see below). A negative binomial distribution (with $r > 1$) is chosen over the geometric distribution ($r = 1$) as it has a mode > 1 (unlike the geometric distribution, the mode of which is always at 1):

$$\operatorname{mode} IP = \begin{cases} \left\lfloor \frac{(1-p)(r-1)}{p} + 1 \right\rfloor, & \text{if } \frac{(1-p)(r-1)}{p} \notin \mathbb{Z},\ r > 1 \\ \frac{(1-p)(r-1)}{p} + 1 \quad \text{and} \quad \frac{(1-p)(r-1)}{p}, & \text{if } \frac{(1-p)(r-1)}{p} \in \mathbb{Z},\ r > 1 \\ 1, & \text{if } r \leq 1, \end{cases}$$

and longitudinal data suggests that the incubation period is generally longer than 1 month [1–7]. We note, however, that using a negative binomial distribution results in slower mixing of the MCMC chain, as it increases the strength of the correlation between the incubation period durations and p [8]. It is not possible to tackle this correlation using a non-centred MCMC algorithm, as is the normal approach for continuous-time models (see [8]), as the monthly time step is large relative to the typical incubation period duration and this results in large discontinuities in the likelihood surface where the incubation periods change, which the non-centred algorithm is unable to navigate. We therefore deal with this issue by running the MCMC chain for a large number of iterations.

2.2 Missing onset and treatment times

The symptom onset and/or treatment times of a number of cases with onset before 2002 (before the prospective part of the study) are missing, and only their year of onset is available. So that the missing onset and treatment times (denoted by $\mathbf{I}'$ and $\mathbf{R}'$), or times from onset to treatment ($\mathbf{OT}' := \mathbf{R}' - \mathbf{I}'$), could be imputed, we fitted a negative binomial distribution with shape parameter r_o and mean μ to the observed onset-to-treatment times via maximum likelihood estimation, such that $\mathbf{OT} = (OT_j) := (R_j - I_j)_{j=1,\dots,n_{IR}} \sim \mathrm{NB}(r_o, \mu)$, where n_{IR} is the total number of cases with observed onset and treatment times. The corresponding probability mass function is

$$g(x) = \mathbb{P}(OT = x) = \frac{\Gamma(r_o + x - 1)}{(x-1)!\,\Gamma(r_o)} \left(\frac{r_o}{r_o + \mu - 1}\right)^{r_o} \left(\frac{\mu - 1}{r_o + \mu - 1}\right)^{x-1}, \quad x \in \{1, 2, 3, \dots\}, \tag{2}$$

and the resulting parameter estimates were $r_o = 2.44$ and $\mu = 4.41$ months. Fig 1 shows the distribution of the observed onset-to-treatment times and the fitted distribution.

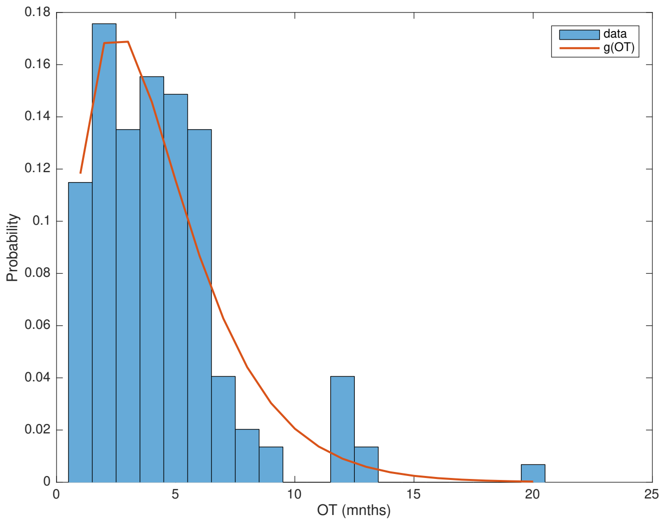

Fig 1. Distribution of observed onset-to-treatment times and fitted negative binomial probability mass function, $g(OT)$.

2.3 Catalytic model for LST data

As described in the main text, we compare the model without any diagnostic data incorporated with a model in which asymptomatic LST-positive individuals are treated as immune, i.e. as being in the recovered state $\mathcal{R}$. We incorporate the LST status data from the annual cross-sectional surveys from 2002-2004 using a variable asymptote catalytic model for the age-prevalence distribution of LST-positivity [9]. The model assumes that a certain proportion $1 - c$ of asymptomatically infected individuals never become LST-positive (due to poor test sensitivity or having too weak a cell-mediated immune response), that the remaining asymptomatically infected individuals become LST-positive with age at a constant rate λ_L, and that those who become LST-positive possess lifelong immunity to VL. We note that lifelong immunity following LST conversion is a relatively strong assumption, since there is some uncertainty over the duration of immunity and its relationship to LST status [10, 11]. Many individuals who were LST-positive in the first and/or second survey had subsequent LST-negative results, but the sensitivity and potency of the leishmanin antigen used in the test decreased during the study suggesting that the true rate of LST loss was much lower [12]. Fitting a catalytic model that included loss of LST positivity to the age-prevalence distribution of asymptomatic LST positivity from the first survey suggested a long average duration of LST positivity (~59 years) with a negligible difference in quality of fit (results not presented). Hence, the assumption of lifelong immunity was deemed an acceptable first approximation, and should not have significantly affected the transmission parameter estimates, given the apparently much shorter timescale of the study than of the wane in immunity.

Denoting age by a and the prevalence of ever LST-positive individuals by p_L, the model can be written as

$$\frac{\mathrm{d}p_L}{\mathrm{d}a} = \lambda_L(c - p_L),$$
$$p_L(0) = 0,$$

the solution of which is

$$p_L(a) = c(1 - e^{-\lambda_L a}).$$

The model was fitted to the asymptomatic LST prevalence data from the 2002 survey only, due to the issues with test sensitivity in the later surveys. The proportion of asymptomatic individuals who

ever become LST-positive and the LST conversion rate were estimated by maximising the likelihood of the observed data, namely

$$L_L(c,\lambda_L) = \prod_{a=3}^{80} (p_L(a))^{k_a}(1-p_L(a))^{n_a-k_a} \tag{3}$$

where n_a is the number of individuals of age a (in years) without previous VL, and k_a is the number of asymptomatic LST-positive individuals of age a.

The maximum likelihood estimates obtained were $\hat{c} = 0.60$ (95% CI 0.50–0.71) and $\hat{\lambda}_L = 0.043\,\text{yr}^{-1}$ (95% CI 0.028–0.058 yr^{-1}) (corresponding to an average age of LST conversion of 23 yrs). The model fit is shown in Fig 2.

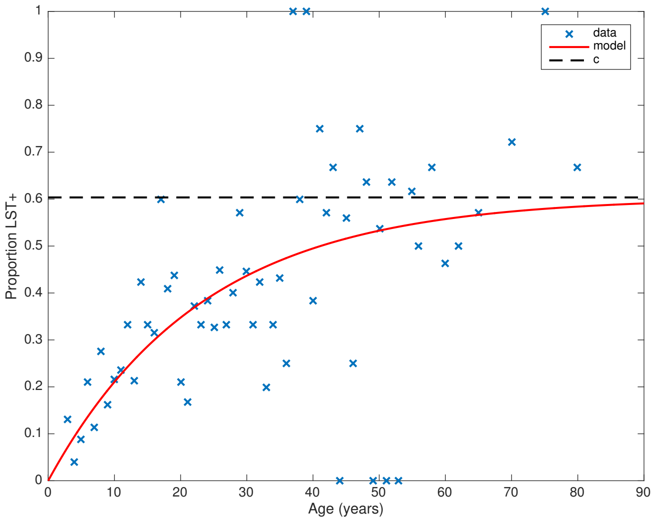

Fig 2. Age-prevalence distribution of LST positivity in 2002 among individuals without any history of VL and fitted catalytic model.

The estimated LST conversion rate was used to incorporate the LST status data into the spatiotemporal model as follows. For individuals who were LST-positive at their first test (at $t = t_L$), the probability that they were already LST-positive in January 1998 ($t = 1$) given their age was calculated as:

$$\mathbb{P}(\text{LST+ in Jan 1998}|\text{LST+ at 1st test}) = \frac{\mathbb{P}(\text{LST+ in Jan 1998})}{\mathbb{P}(\text{LST+ at 1st test})} = \frac{p_L(a_1)}{p_L(a_{t_L})}, \tag{4}$$

where a_1 and a_{t_L} denote their ages in January 1998 and at their first skin test. These individuals were then assigned an initial LST status (positive or negative) according to the calculated probabilities. LST conversion times for the initially LST-negative individuals were drawn from $\text{Exp}(\lambda_L)$ conditional on the conversion time being before the first skin test. For individuals who became LST-positive between surveys, the time after the negative LST test at which they converted was also drawn from $\text{Exp}(\lambda_L)$, conditional on the conversion having happened before the positive test. All initially LST-positive individuals and individuals who became LST-positive during the study were treated as being in state $\mathcal{R}$ from January 1998 or their month of conversion, respectively, for the duration of the study.

2.4 Complete data likelihood

We assume that the whole of the wave of VL incidence in the village was observed, i.e. that all VL cases with onset between January 1999 and June 2004 were recorded and that there were no

individuals infected during the study period who developed symptoms after the study. Given the long incubation period of VL, the latter part of this assumption may be inaccurate, but it greatly simplifies the parameter inference process.

As described under Methods in the main text, the likelihood of each individual's infection history, conditional on those of all other individuals and the parameter values $\boldsymbol{\theta}$, can be calculated by multiplying together their monthly probabilities of avoiding infection up to the month they were infected or the end of the study if they did not get VL. Hence, using the notation defined in the Methods section in the main text, the contribution of an individual i who remained susceptible throughout the whole period (from $t = 1$ to $t = T$) to the overall likelihood is

$$Q_i(T) := \mathbb{P}(E_i > T|\mathbf{A}, \boldsymbol{\theta}) = \prod_{t=1}^{T} q_i(t), \tag{5}$$

while that of an individual j who was infected in month $t_j < T$ is

$$P_j(t_j) := \mathbb{P}(E_j = t_j|\mathbf{A}, \boldsymbol{\theta}) = \left(\prod_{t=1}^{t_j-1} q_j(t)\right) p_j(t_j), \tag{6}$$

If all infection times $\mathbf{E} = (E_j)_{j=1,\ldots,n_I}$, symptom onsets $\mathbf{I} = (I_j)_{j=1,\ldots,n_I}$, and recovery times $\mathbf{R} = (R_j)_{j=1,\ldots,n_I}$ had been observed (where n_I denotes the total number of cases), the overall likelihood would just be the product of these contributions over all individuals, since each infection is assumed to have occurred independently given the history of the epidemic up to that point

$$L(\boldsymbol{\theta}; \mathbf{A}) := \mathbb{P}(\mathbf{A}|\boldsymbol{\theta}) = \prod_{i \in \mathcal{S}(T)} Q_i(T) \prod_{j=1}^{n_I} P_j(t_j), \tag{7}$$

where $\mathbf{A} = (\mathbf{E}, \mathbf{I}, \mathbf{R})$, which can be rewritten as a product over months 1 to T of all avoidance probabilities and infection events

$$L(\boldsymbol{\theta}; \mathbf{A}) = \prod_{t=1}^{T} \left(\prod_{i \in \mathcal{S}(t)} q_i(t) \prod_{j \in \{E_j = t\}} p_j(t) \right) \tag{8}$$

However, none of the infection times were observed, and some of the symptom onset times and recovery times for pre-2002 cases were not recorded, so this must be accounted for in the likelihood. Conditional on being infected, the durations of individuals' incubation periods prior to symptoms are determined by Eq (1), and the durations of symptoms for individuals missing either their onset or treatment time, or both, are described by Eq (2). Thus, the full likelihood for the data is

$$L(\boldsymbol{\theta}; \mathbf{A}) = \underbrace{\prod_{t=1}^{T} \left(\prod_{i \in \mathcal{S}(t)} q_i(t) \prod_{j \in \{E_j = t\}} p_j(t) \right) \prod_{k=1}^{n_I} f(I_k - E_k)}_{L_1} \underbrace{\prod_{l=1}^{n'_I + n'_R + n'_{IR}} g(R_l - I_l)}_{L_2}, \tag{9}$$

where $n'_I = 1$, $n'_R = 2$ and $n'_{IR} = 30$ are the numbers of cases missing onset, treatment, and both onset and treatment times respectively.

We note that, unlike many previous authors [13–17], we do not condition the likelihood on the first infection time, $E_{\min} = \min_{j \in \{1,\ldots,n_I\}}(E_j)$. We instead assume that the index VL case arose from background transmission, since there were no recorded cases in any of the study paras in 1998. We also assume that the earliest possible infection time of any of the cases was January 1998 (and set this as $t = 1$). Hence the likelihood (Eq (9)) is evaluated over all cases and all months from January 1998 ($t = 1$) to June 2004 ($t = T = 78$).

3 Parameter estimation

3.1 Bayesian inference

If all the infection, onset and recovery times had been observed it would be relatively straightforward to estimate the transmission parameters using standard likelihood-based inference techniques, since the overall likelihood of the epidemic process could be calculated using Eq (7). However, since the infection times are unknown, and some onset and recovery times ($\mathbf{I}'$ and $\mathbf{R}'$) are missing, calculating the likelihood in Eq (7) would require summing over all possible configurations of the missing data $\mathbf{U} = (\mathbf{E}, \mathbf{I}', \mathbf{R}')$, which is computationally infeasible. Hence, we treat the infection times and missing onset and recovery times as extra parameters and use a data augmentation approach to sample from the joint posterior distribution of the model parameters $\boldsymbol{\theta} = (\beta, \alpha, \epsilon, \delta, p)$ and the missing data given the observed data $\mathbf{Y} = (\tilde{\mathbf{I}}, \tilde{\mathbf{R}}, \mathbf{I}_R, \mathbf{R}_R)$

$$\mathbb{P}(\boldsymbol{\theta}, \mathbf{U}|\mathbf{Y}) = \frac{\mathbb{P}(\mathbf{Y}, \mathbf{U}|\boldsymbol{\theta})\mathbb{P}(\boldsymbol{\theta})}{\mathbb{P}(\mathbf{Y})} \propto L(\mathbf{A}; \boldsymbol{\theta})\mathbb{P}(\boldsymbol{\theta}). \tag{10}$$

We do this using a 'centred' MCMC data augmentation algorithm in which we iterate between sampling from the conditional posterior distribution of the parameters given the observed data and the current value of the missing data, $\mathbb{P}(\boldsymbol{\theta}|\mathbf{U}, \mathbf{Y})$, and the conditional posterior distribution of the missing data given the current parameter values and the observed data, $\mathbb{P}(\mathbf{U}|\boldsymbol{\theta}, \mathbf{Y})$ (see [8] §§1.7, 2.2.3, 2.3 and 2.6 for a full discussion of centred algorithms).

3.2 MCMC data augmentation scheme

The MCMC algorithm used to sample from the joint posterior distribution of the parameters and the missing data (Eq (10)) is similar to 'Centred Algorithm I' in §2.2.3 of [8] and a centred version of the algorithm described in [18], but with some modifications since the model is in discrete rather than continuous time, and there are missing onset and treatment times as well as unobserved infection times. The algorithm also accounts for the fact that some individuals were born or died during the study when updating the unknown infection times and missing onset and treatment times (using the birth/death times as bounds on the proposed unobserved times), but we omit these details from the following description for simplicity.

3.2.1 Initial parameter values and missing data values

In order to run the MCMC algorithm, initial values for the parameters $\boldsymbol{\theta}$ and missing data $\mathbf{U}$ (infection times and missing onset and treatment times), and prior distributions for the parameters must be chosen. A range of initial parameter values within plausible bounds were chosen to test the convergence of the MCMC, and initial values close to the values identified from these runs ($\boldsymbol{\theta}_0 = (\beta_0, \alpha_0, \epsilon_0, \delta_0, p_0) = (0.2, 50, 0.001, 0.001, 3/7)$) used for the final MCMC runs to minimise the burn-in time (the number of iterations required for the chain to converge). The initial infection times $\mathbf{E}_0$ and missing onset and treatment times, $\mathbf{I}'_0$ and $\mathbf{R}'_0$, were drawn using the following steps:

1. For cases for whom only the onset year is known, the onset month was drawn uniformly at random from the onset year (i.e. from a discrete uniform distribution $\mathrm{U}(1, 12)$).
2. For cases with missing treatment times, an onset-to-treatment time was drawn from $OT'_0 \sim \mathrm{NB}(r_o, \mu)$ and added to their onset time, i.e.

$$\mathbf{R}'_0 = \mathbf{I} + \mathbf{OT}'_0 \tag{11}$$

3. For cases with missing onset times but observed treatment times, an onset-to-treatment time was drawn as in 2 and subtracted from their treatment time, i.e.
$$\mathbf{I}'_0 = \mathbf{R} - \mathbf{OT}'_0 \tag{12}$$
4. For all cases, incubation periods were drawn from $IP \sim \mathrm{NB}(r, p_0)$ and subtracted from their onset times
$$\mathbf{E}_0 = \mathbf{I} - \mathbf{IP}_0. \tag{13}$$

3.2.2 Prior distributions

The prior distributions for the parameters $\boldsymbol{\theta}$ were chosen as described in the main text. A Beta(a,b) distribution was chosen as the prior for p, since it is conjugate to the likelihood, i.e. yields a beta distribution for the posterior distribution of p given the values of the other parameters $\boldsymbol{\beta} = (\beta, \alpha, \epsilon, \delta)$ and the data:
$$(p|\boldsymbol{\beta}, \mathbf{U}, \mathbf{Y}) \sim \mathrm{Beta}\left(a + rn_I, b + \sum_{j=1}^{n_I} IP_j - n_I\right), \tag{14}$$
and means that p can be updated in the MCMC using a Gibbs sampler (i.e. by drawing from this conditional distribution), which is more efficient than using Metropolis-Hastings updates.

3.2.3 MCMC Algorithm

With the initial parameter and missing data values and prior distributions specified as above, the algorithm consists of repeating the following steps:

1. **Update transmission parameters:**
   Update the transmission parameters $\boldsymbol{\beta}|p, \mathbf{U}, \mathbf{Y}$ using an adaptive random walk Metropolis-Hastings step:
   (a) Propose new values $\boldsymbol{\beta}'$ as described in §3.2.4 below.
   (b) Accept $\boldsymbol{\beta}'$ with probability
   $$\min\left(\frac{L_1(\boldsymbol{\beta}')}{L_1(\boldsymbol{\beta})}\frac{\mathbb{P}(\boldsymbol{\beta}')}{\mathbb{P}(\boldsymbol{\beta})}, 1\right).$$
2. **Update incubation period distribution parameter:**
   Gibbs update p by drawing from $\mathbb{P}(p|\boldsymbol{\beta}, \mathbf{U}, \mathbf{Y})$ (Eq (14)).
3. **Move infection times:**
   Update one by one the infection times of a random 20% of cases for whom both the onset and treatment times were observed, and the infection times of all cases missing their onset and/or treatment time:
   (a) For each case j, propose a new infection time E'_j using an independence sampler, i.e. propose a new incubation period $IP'_j \sim \mathrm{NB}(r, p)$ and subtract this from the onset time
   $$E'_j = I_j - IP_j.$$
   (b) Accept the infection time move with probability
   $$\min\left(\frac{L_1(E'_j|\mathbf{E}_{-j}, \mathbf{I}, \mathbf{R}, \boldsymbol{\theta})}{L_1(E_j|\mathbf{E}_{-j}, \mathbf{I}, \mathbf{R}, \boldsymbol{\theta})}\frac{q(IP'_j, IP_j)}{q(IP_j, IP'_j)}, 1\right).$$
   where $q(IP_j, IP'_j) \equiv f(IP'_j)$ is the proposal distribution for the change in the incubation period from IP_j to IP'_j (i.e. the infection time move) and $\mathbf{E}_{-j} = (E_1, \ldots, E_{j-1}, E_{j+1}, \ldots, E_{n_I})$.

4. **Update missing onset times:**
Update one at a time the onset times of all cases whose onset was unobserved (including those with unobserved treatment):

   (a) For each case k, propose a new onset time I'_k, conditional on $I'_k > E_k$ and I'_k being in k's onset year, by drawing a new onset-to-treatment time $OT'_k \sim \mathrm{NB}(r_o, \mu)$ and subtracting it from the treatment time R_k
$$I'_k = R_k - OT'_k.$$

   (b) If $I'_k \leq E_k$ or I'_k is not in k's onset year, reject immediately. Otherwise accept the new onset time with probability
$$\min\left(\frac{L_1(I'_k|\mathbf{I}_{-k}, \mathbf{E}, \mathbf{R}, \boldsymbol{\theta})}{L_1(I_k|\mathbf{I}_{-k}, \mathbf{E}, \mathbf{R}, \boldsymbol{\theta})}, 1\right),$$
where $\mathbf{I}_{-k} = (I_1, \ldots, I_{k-1}, I_{k+1}, \ldots, I_{n_I})$.

5. **Move infection-to-treatment period:**
Update the infection, onset and treatment times of all cases missing both onset and treatment times, one case at a time:

   (a) For case l, propose new infection, onset and treatment times, E'_l, I'_l and R'_l, conditional on I'_l being in l's onset year and $R_l < T$, by drawing a number $M = \mathrm{round}(\mathrm{N}(0, 4)) \neq 0$ and adding this to the current times
$$E'_l = E_l + M,$$
$$I'_l = I_l + M,$$
$$R'_l = R_l + M.$$

   (b) Accept the new times with probability
$$\min\left(\frac{L_1(E'_l, I'_l, R'_l|\mathbf{E}_{-l}, \mathbf{I}_{-l}, \mathbf{R}_{-l}, \boldsymbol{\theta})}{L_1(E_l, I_l, R_l|\mathbf{E}_{-l}, \mathbf{I}_{-l}, \mathbf{R}_{-l}, \boldsymbol{\theta})}, 1\right),$$
where $\mathbf{R}_{-l} = (R_1, \ldots, R_{l-1}, R_{l+1}, \ldots, R_{n_I})$.

6. **Update missing treatment times:**
Update the treatment times of all cases missing their treatment times but with known onset times, one by one:

   (a) Propose a new treatment time R'_m as:
$$R'_m = I_m + OT'_m$$
where $OT'_m \sim \mathrm{NB}(r_o, \mu)$.

   (b) Accept the new treatment time with probability
$$\min\left(\frac{L_1(R'_m|\mathbf{E}, \mathbf{I}, \mathbf{R}_{-m}, \boldsymbol{\theta})}{L_1(R_m|\mathbf{E}, \mathbf{I}, \mathbf{R}_{-m}, \boldsymbol{\theta})}, 1\right).$$

Note that we use only the L_1 part of the likelihood expression in Eq (9) in the acceptance probabilities for the different steps, since we propose new onset-to-treatment times from $\mathrm{NB}(r_o, \mu)$ for individuals missing their onset and/or treatment time, so the L_2 term for the probability of the onset-to-treatment durations cancels with the proposal distribution ratio in steps 4 and 6 and is unaffected by the other updates. Updating 20% of infection times of cases with observed onset and

treatment times, and all infection times of individuals missing their onset and/or treatment time in each MCMC iteration ensures good mixing of the MCMC chain while avoiding it becoming prohibitively slow to run. In order to explore the high-dimensional parameter space (of the transmission parameters and missing data) thoroughly, the algorithm was run for $N = 400,000$ iterations (i.e. steps 1–6 were repeated 400,000 times), including a burn-in of 4000 iterations which was discarded.

3.2.4 Adaptive random walk Metropolis-Hastings

Rather than individually updating the transmission parameters $\boldsymbol{\beta} = (\beta, \alpha, \epsilon, \delta)$ in step 1 of the algorithm, which would make the algorithm slow due to the computational expense of recalculating the likelihood, we block update them. Following the work of Haario et al [19] and Roberts and Rosenthal [20], we use an adaptive proposal density to save having to manually tune the proposal variances for the parameters. The multivariate normal proposal density at the kth iteration is given by:

$$q_k(\boldsymbol{\beta}_k, \boldsymbol{\beta}_{k+1}) = \begin{cases} \mathrm{N}(\boldsymbol{\beta}_k, (0.1)^2\boldsymbol{\Sigma}_0/n_{\boldsymbol{\beta}}), & \text{for } k \leq 100 \\ (1-\xi)\mathrm{N}(\boldsymbol{\beta}_k, (2.38)^2\boldsymbol{\Sigma}_k/n_{\boldsymbol{\beta}}) + \xi\mathrm{N}(\boldsymbol{\beta}_k, (0.1)^2\boldsymbol{\Sigma}_0/n_{\boldsymbol{\beta}}), & \text{for } k > 100, \end{cases} \tag{15}$$

where $\boldsymbol{\Sigma}_0$ is an initial guess for the parameter covariance matrix (e.g. $\boldsymbol{\Sigma}_0 = \mathrm{diag}(\beta_0^2, \alpha_0^2, \epsilon_0^2, \delta_0^2)$), $n_{\boldsymbol{\beta}}$ is the number of transmission parameters being updated, $\boldsymbol{\Sigma}_k$ is the current running estimate of the $n_{\boldsymbol{\beta}}$-dimensional covariance matrix of the posterior density (the covariance of the samples in the chain up to the kth iteration), and ξ is a small positive constant, which we take to be 0.05 [20]. This means that, for $k > 100$, 95% of proposals are made from $\mathrm{N}(\boldsymbol{\beta}_k, (2.38)^2\boldsymbol{\Sigma}_k/n_{\boldsymbol{\beta}})$, and the other 5% of proposals are drawn from $\mathrm{N}(\boldsymbol{\beta}_k, (0.1)^2\boldsymbol{\Sigma}_0/n_{\boldsymbol{\beta}})$ to ensure the chain does not get stuck in regions of parameter space away from the highest posterior density, e.g. due to singular values of $\boldsymbol{\Sigma}_k$. The proposal density thus adapts for optimal scaling as the chain converges.

4 Model comparison

4.1 Deviance information criterion for missing data models

When all variables in a model are fully observed, i.e. there is no missing data, the deviance information criterion (DIC) is defined as

$$\begin{aligned} \mathrm{DIC} &= D(\tilde{\boldsymbol{\theta}}) + 2p_D \\ &= \overline{D(\boldsymbol{\theta})} + p_D, \end{aligned} \tag{16}$$

where $D(\boldsymbol{\theta})$ is the deviance, given (up to an additive constant dependent only on the data) by

$$D(\boldsymbol{\theta}) = -2\log(L(\boldsymbol{\theta}; \mathbf{A})); \tag{17}$$

p_D is the effective number of parameters in the model, defined by

$$p_D = \overline{D(\boldsymbol{\theta})} - D(\tilde{\boldsymbol{\theta}}), \tag{18}$$

where $\overline{D(\boldsymbol{\theta})} = \mathbb{E}_{\boldsymbol{\theta}}[D(\boldsymbol{\theta})]$ is the posterior mean deviance; and $\tilde{\boldsymbol{\theta}}$ is a point estimate of $\boldsymbol{\theta}$ from its posterior distribution (such as the posterior mean or mode) [21]. $\overline{D(\boldsymbol{\theta})}$ is a measure of how well the model fits the data, and p_D is a penalty term for the complexity of the model (which increases with the number of model parameters), so models with lower DIC values are preferred.

However, since the epidemic process was not fully observed, we require a version of DIC that accounts for some data being missing. Following Celeux et al [22], we use a version of DIC for

missing data models based on the complete data likelihood $L(\boldsymbol{\theta};\mathbf{A})=\mathbb{P}(\mathbf{U},\mathbf{Y}|\boldsymbol{\theta})$

$$\begin{aligned}\mathrm{DIC}&=\overline{D(\boldsymbol{\theta})}+p_D\\&=2\overline{D(\boldsymbol{\theta})}-D(\tilde{\boldsymbol{\theta}})\\&=-4\mathbb{E}_{\boldsymbol{\theta},\mathbf{U}}[\log L(\boldsymbol{\theta};\mathbf{U},\mathbf{Y})]+2\mathbb{E}_{\mathbf{U}}[\log L(\tilde{\boldsymbol{\theta}};\mathbf{U},\mathbf{Y})].\end{aligned}\tag{19}$$

Note that this is the same as the DIC for fully observed data in Eq (16), except that we have taken the expectation over the missing data $\mathbf{U}$. This DIC is equivalent to DIC_4 in [22] except that we use the posterior mode for $\tilde{\boldsymbol{\theta}}$ rather than the posterior mean, due to non-normality in the posterior distribution.

The first term in Eq (19) can be straightforwardly estimated as the mean of the values of the log-likelihood over the MCMC samples:

$$\mathbb{E}_{\boldsymbol{\theta},\mathbf{U}}[\log L(\boldsymbol{\theta};\mathbf{U},\mathbf{Y})]\approx\frac{1}{N}\sum_{i=1}^{N}\log L(\boldsymbol{\theta}_i;\mathbf{U}_i,\mathbf{Y}).$$

The second term in Eq (19) is calculated by storing the values of the missing data while running the MCMC, re-running the full log-likelihood computation for each iteration using the stored values and the posterior mode, and averaging these values

$$\mathbb{E}_{\mathbf{U}}[\log L(\tilde{\boldsymbol{\theta}};\mathbf{U},\mathbf{Y})]\approx\frac{1}{N}\sum_{i=1}^{N}\log L(\boldsymbol{\theta}_{\mathrm{mode}};\mathbf{U}_i,\mathbf{Y}).$$

5 Further details on results

5.1 MCMC output

The output of the MCMC scheme for the exponential kernel model, in terms of the log-likelihood and model parameters, is shown in Fig 6 in the main text. The corresponding autocorrelations for the transmission parameters (β,α,ϵ) and incubation period distribution parameter p are shown in Fig 3. The trace plot for the log-likelihood and roughly Gaussian-shaped histograms in Fig 6 (main text), and the autocorrelation plots in Fig 3 demonstrate that the MCMC algorithm mixed well and explored the space of the model parameters thoroughly. The high autocorrelation for p is due to the strong correlation between the incubation period durations and p and the difficulty of exploring this correlation efficiently for a discrete time model with large time steps. The acceptance rates for Step 1 of the MCMC algorithm (the adaptive Metropolis-Hastings update of the transmission parameters) were between 27.6% and 35.5% for all models with more than one transmission parameter, i.e. well within the ideal acceptance rate range of 15–50% for models with multiple parameters [23]. The corresponding acceptance rates for Steps 3–6 of the algorithm (the missing infection, onset and treatment time updates) were between 70.8% and 86.2%, indicating efficient exploration of the high-dimensional space of missing data.

5.1.1 Unobserved infection, onset and treatment times

Fig 4 shows the posterior distributions of the unobserved infection times, and onset and treatment times when missing, for a selection of cases. The first row of plots is for cases with known onset and treatment times and shows that for some cases there is sufficient information in the data to constrain their probable infection time, e.g. the first plot is for an individual with onset at $I_{15}=73$ months, and it shows that their most likely infection time was $E_{15}=69$ months. The second row of plots is for cases with known onset time but missing treatment time or vice versa. For those missing their treatment time (left-hand two plots), the posterior distributions for the infection times and treatment times closely follow the prior distributions, suggesting that their is little additional information in

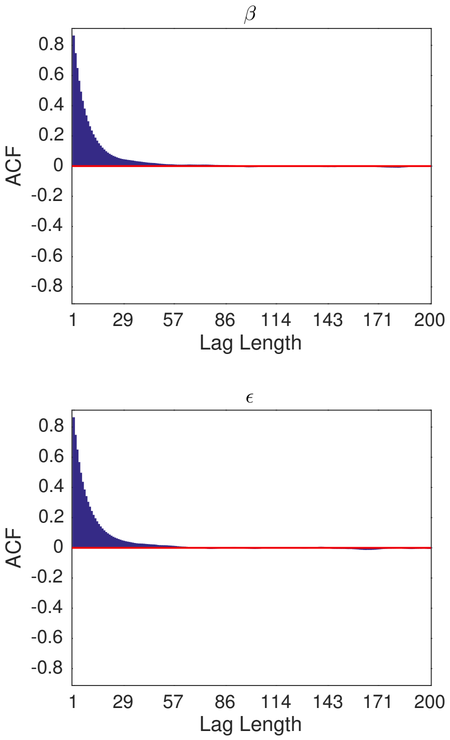

Fig 3. Autocorrelation plots for the model parameters $\boldsymbol{\theta} = (\beta, \alpha, \epsilon, p)$ for the exponential kernel model, showing the correlation between samples in the MCMC chain at different lag lengths (different numbers of iterations apart).

the data about when these individuals were infected and treated. Posterior distributions for the infection, onset and treatment times for three cases for whom only the onset year is known are shown in the final row. The posterior distributions for the infection and treatment times no longer have the shape of the prior distributions due to the unknown onset time, and for most cases appear to be constrained only by the onset time having to be in the onset year, although the different shapes of the distributions suggest that the data is informative about the unobserved times for some cases.

5.1.2 Parameter correlation

Correlations between the transmission and incubation period distribution parameters were examined by plotting heatmaps of the posterior density for each pair of parameters and calculating the parameter correlation coefficient matrix of the MCMC samples (where the correlation coefficient $\rho(\mathbf{x}, \mathbf{y})$ of two vectors of samples $\mathbf{x}$ and $\mathbf{y}$ is defined by $\rho(\mathbf{x}, \mathbf{y}) = \mathrm{cov}(\mathbf{x}, \mathbf{y})/\sqrt{\mathrm{var}(\mathbf{x})\mathrm{var}(\mathbf{y})}$ and $\mathrm{cov}(\mathbf{x}, \mathbf{y})$ is the covariance of $\mathbf{x}$ and $\mathbf{y}$ and $\mathrm{var}(\mathbf{x})$ is the variance of $\mathbf{x}$, such that $\rho = 1$ represents perfect positive correlation, $\rho = -1$ perfect negative correlation, and $\rho = 0$ no correlation). Fig 5 shows the marginal pairwise posterior densities for the exponential kernel model, and the corresponding parameter correlation coefficient matrix is

$$\boldsymbol{\rho}(\boldsymbol{\theta}, \boldsymbol{\theta}) = \begin{array}{c} \\ \end{array} \begin{matrix} \beta & \alpha & \epsilon & p \end{matrix} \\ \begin{pmatrix} 1 & 0.25 & -0.47 & 0.04 \\ 0.25 & 1 & -0.30 & 0.14 \\ -0.47 & -0.30 & 1 & -0.02 \\ 0.04 & 0.14 & -0.02 & 1 \end{pmatrix} \begin{matrix} \beta \\ \alpha \\ \epsilon \\ p \end{matrix}$$

The strongest correlation is between the rate constant for transmission from VL cases, β, and the

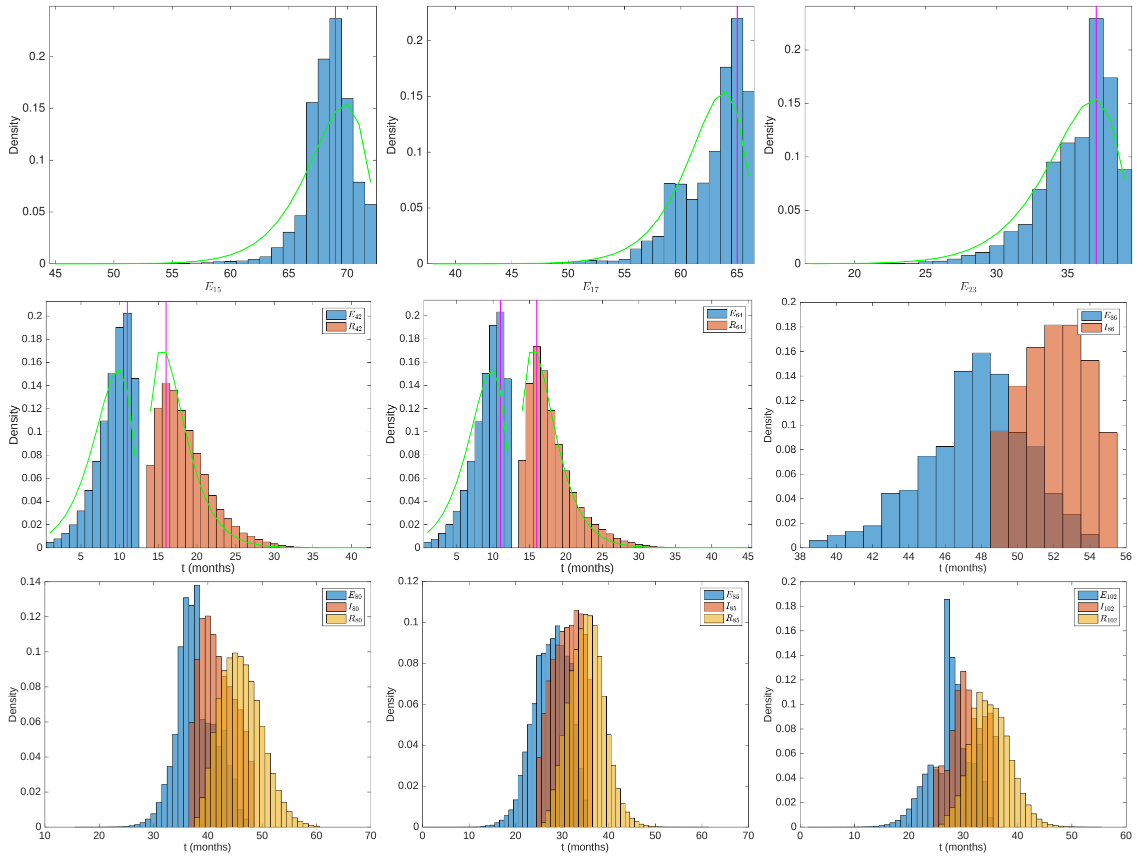

Fig 4. Posterior distributions of the unobserved infection, onset and treatment times of a selection of cases. First row: Posterior distributions of the infection times of cases with known onset times (blue histograms), with prior distributions (green lines) and modes (magenta lines). Second row: Posterior distributions of infection and treatment/onset times for cases with known onset time and missing treatment time or vice versa. Last row: Posterior distributions of infection, onset and treatment times for cases with only known onset year.

constant background transmission rate, ϵ, which are negatively correlated. This makes intuitive sense, as the greater the proportion of transmission that is accounted for by proximity to VL cases (the higher the value of β), the less needs to be accounted for by the background transmission (the lower ϵ will be). The risk decay distance α is negatively correlated with the background transmission rate, since greater values of α correspond to a slower decrease in risk with distance, such that fewer cases need to be explained by background transmission. There is weak positive correlation between α and p, i.e. between a slower decrease in risk with distance and shorter incubation periods, because a flatter spatial kernel increases the chances that a case was infected by a case further away who was infectious nearer to their onset time.

5.2 Sensitivity analysis for pre-symptomatic infectiousness, h_0

Since the relative infectiousness of pre-symptomatic individuals, h_0, is unknown, we fitted the exponential kernel model (with and without additional within-household transmission) for different fixed values of h_0 (0, 0.03, 0.1, 0.5) to test the sensitivity of the parameter estimates to h_0. The results are shown in Table 1.

The posterior mode for α remains fairly constant as h_0 is increased, but the modes for β and ϵ both decrease, both with and without additional within-household transmission. The estimated

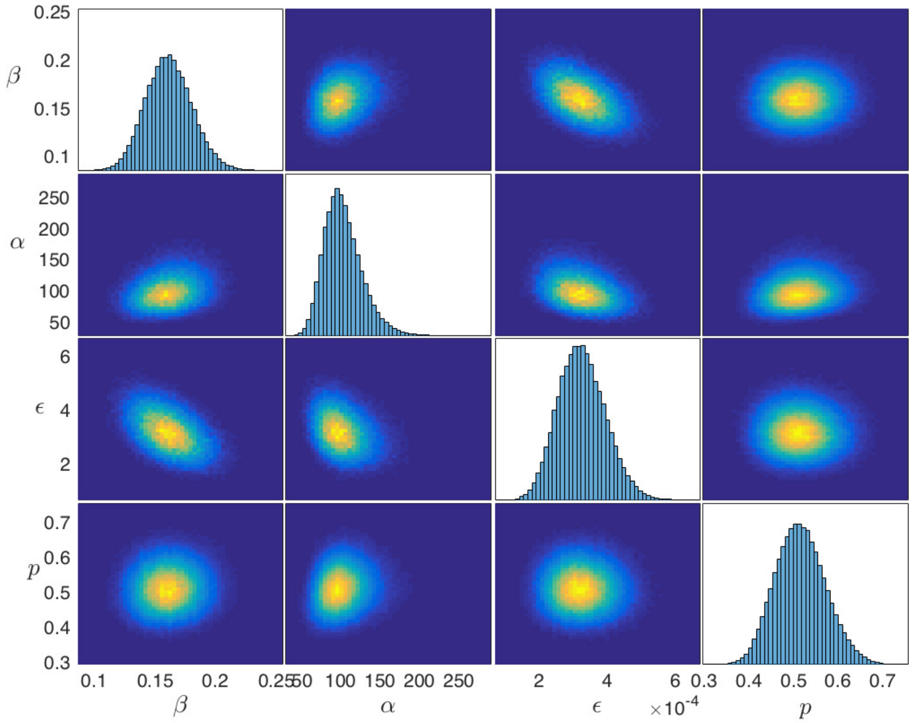

Fig 5. Posterior distributions and correlations of the transmission parameters for the exponential kernel model. Main diagonal: Marginal posterior distributions of the transmission parameters. Off diagonals: Heatmap plots of pairwise marginal posterior densities showing the correlations between parameters. Lighter yellower colours indicate areas of higher posterior density.

incubation period distribution parameter p is very similar for all values of h_0 (posterior modes 0.49–0.51, 95% HPDIs (0.39–0.61)–(0.41–0.63)). Negative correlation between h_0 and each of β and ϵ is expected given a constant spatial kernel and incubation period distribution, as increasing h_0 with the other parameters fixed increases the contribution of pre-symptomatic individuals. According to the DICs for the different h_0 values, the best-fitting model is that with additional within-household transmission in which pre-symptomatic individuals are uninfectious ($h_0 = 0$). However, the differences in DIC between this model and those without additional within-household transmission and either uninfectious or slightly infectious ($h_0 = 0.03$) pre-symptomatics are small (ΔDIC < 8), indicating that there is very little difference in the goodness-of-fit of these models. Thus, in the main text, we continue with our initial assumption from previous modelling estimates that pre-symptomatics are slightly infectious compared to symptomatic individuals ($h_0 = 0.03$), and focus on the results of the exponential kernel model (which is the best-fitting model for $h_0 = 0.03$).

Table 1. Parameter estimates and DIC for different pre-symptomatic infectiousnesses for the exponential kernel model.

**Pre-symptomatic relative infectiousness, h_0**	**Parameter estimates*** β (mnth^{-1})	95% HPDI	α (m)	95% HPDI	ϵ ($\times 10^{-4}$ mnth^{-1})	95% HPDI	δ ($\times 10^{-3}$ mnth^{-1})	95% HPDI	DIC
0	0.17	(0.13–0.20)	96	(61–153)	3.1	(2.0–4.6)	$0^{\dagger}$	-	3456.9
0.03	0.16	(0.13–0.20)	95	(59–152)	3.3	(2.0–4.6)	$0^{\dagger}$	-	3459.5
0.10	0.15	(0.12–0.19)	95	(61–151)	3.0	(1.8–4.4)	$0^{\dagger}$	-	3469.7
0.50	0.13	(0.10–0.15)	95	(61–146)	2.5	(1.3–3.9)	$0^{\dagger}$	-	3480.6
Additional within-household transmission									
0	0.16	(0.12–0.20)	106	(66–169)	3.1	(2.1–4.7)	1.6	(0.1–4.4)	3452.2
0.03	0.15	(0.12–0.19)	102	(62–160)	3.1	(1.9–4.5)	1.4	(0.1–4.4)	3459.7
0.10	0.15	(0.11–0.18)	97	(62–162)	2.9	(1.8–4.4)	1.8	(0.1–4.4)	3466.5
0.50	0.12	(0.09–0.15)	100	(69–162)	2.4	(1.3–3.8)	1.6	(0.1–3.8)	3474.4

* Modes of marginal posterior distributions for parameters and 95% highest posterior density intervals (HPDIs).

† Assumed.

References

1. Ostyn B, Gidwani K, Khanal B, Picado A, Chappuis F, Singh SP, et al. Incidence of symptomatic and asymptomatic Leishmania donovani infections in High-Endemic foci in India and Nepal: A prospective study. PLoS Neglected Tropical Diseases. 2011;5(10):1–7. doi:10.1371/journal.pntd.0001284.

2. Topno RK, Das VNR, Ranjan A, Pandey K, Singh D, Kumar N, et al. Asymptomatic infection with visceral leishmaniasis in a disease-endemic area in Bihar, India. American Journal of Tropical Medicine and Hygiene. 2010;83(3):502–506. doi:10.4269/ajtmh.2010.09-0345.

3. Das VNR, Siddiqui Na, Verma RB, Topno RK, Singh D, Das S, et al. Asymptomatic infection of visceral leishmaniasis in hyperendemic areas of Vaishali district, Bihar, India: A challenge to kala-azar elimination programmes. Transactions of the Royal Society of Tropical Medicine and Hygiene. 2011;105(11):661–666. doi:10.1016/j.trstmh.2011.08.005.

4. Bimal S, Das VNR, Sinha PK, Gupta AK, Verma N, Ranjan A, et al. Usefulness of the direct agglutination test in the early detection of subclinical Leishmania donovani infection: a community-based study. Annals of Tropical Medicine & Parasitology. 2005;99(8):743–749. doi:10.1179/136485905X65107.

5. Singh S, Kumari V, Singh N. Predicting Kala-Azar Disease Manifestations in Asymptomatic Patients with Latent Leishmania donovani Infection by Detection of Antibody against Recombinant K39 Antigen. Clinical and Diagnostic Laboratory Immunology. 2002;9(3):568–572. doi:10.1128/CDLI.9.3.568.

6. Saha P, Ganguly S, Chatterjee M, Das SB, Kundu K, Guha SK, et al. Asymptomatic leishmaniasis in kala-azar endemic areas of Malda district, West Bengal, India. PLoS Neglected Tropical Diseases. 2017;11(2):e0005391. doi:10.1371/journal.pntd.0005391.

7. Gidwani K, Kumar R, Rai M, Sundar S. Longitudinal Seroepidemiologic Study of Visceral Leishmaniasis in Hyperendemic Regions of Bihar, India. American Journal of Tropical Medicine and Hygiene. 2009;80(3):345–346.

8. Kypraios T. Efficient Bayesian Inference for Partially Observed Stochastic Epidemics and A New Class of Semi−Parametric Time Series Models. Lancaster University; 2007. Available from: http://eprints.lancs.ac.uk/26392/1/kypraios.pdf.

9. Vynnycky E, White R. An introduction to infectious disease modelling. Oxford University Press; 2010.

10. Le Rutte EA, Chapman LAC, Coffeng LE, Jervis S, Hasker EC, Dwivedi S, et al. Elimination of visceral leishmaniasis in the Indian subcontinent: a comparison of predictions from three transmission models. Epidemics. 2017;18:67–80. doi:10.1016/j.epidem.2017.01.002.

11. Chapman LAC, Morgan ALK, Adams ER, Bern C, Medley GF, Hollingsworth TD. Age trends in asymptomatic and symptomatic Leishmania donovani infection in the Indian subcontinent: a review of data from diagnostic and epidemiological studies. 2018;doi:10.1371/journal.pntd.0006803.

12. Bern C, Amann J, Haque R, Chowdhury R, Ali M, Kurkjian KM, et al. Loss of leishmanin skin test antigen sensitivity and potency in a longitudinal study of visceral leishmaniasis in Bangladesh. American Journal of Tropical Medicine and Hygiene. 2006;75(4):744–8.

13. O'Neill PD, Roberts GO. Bayesian inference for partially observed stochastic epidemics. Journal of the Royal Statistical Society A. 1999;162:121–129.

14. Britton T, O'Neill PD. Bayesian Inference for Stochastic Epidemics in Populations with Random Social Structure. Scandinavian Journal of Statistics. 2002;29(1998):375–390.

15. Neal PJ, Roberts GO. Statistical inference and model selection for the 1861 Hagelloch measles epidemic. Biostatistics. 2004;5(2):249–261.

16. Jewell CP, Keeling MJ, Roberts GO. Predicting undetected infections during the 2007 foot-and-mouth disease outbreak. Journal of the Royal Society Interface. 2009;6:1145–1151. doi:10.1098/rsif.2008.0433.

17. Jewell CP, Kypraios T, Christley RM, Roberts GO. A novel approach to real-time risk prediction for emerging infectious diseases: A case study in Avian Influenza H5N1. Preventive Veterinary Medicine. 2009;91:19–28. doi:10.1016/j.prevetmed.2009.05.019.

18. Jewell CP, Kypraios T, Neal P, Roberts GO. Bayesian Analysis for Emerging Infectious Diseases. Bayesian Analysis. 2009;4(4):465–496. doi:10.1214/09-BA417.

19. Haario H, Saksman E, Tamminen J. An Adaptive Metropolis Algorithm. Bernoulli. 2001;7(2):223. doi:10.2307/3318737.

20. Roberts GO, Rosenthal JS. Examples of Adaptive MCMC. Journal of Computational and Graphical Statistics. 2009;18(2):349–367. doi:10.1198/jcgs.2009.06134.

21. Spiegelhalter DJ, Best NG, Carlin BP, van der Linde A. Bayesian Measures of Model Complexity and Fit. Journal of the Royal Statistical Society Series B (Statistical Methodology). 2002;64(4):583–639. doi:10.1111/1467-9868.00353.

22. Celeux G, Forbes F. Deviance information criteria for missing data models. Bayesian Analysis. 2006;1(4):651–673. doi:10.1214/06-BA122.

23. Roberts GO, Rosenthal JS. Optimal scaling for various Metropolis-Hastings algorithms. Statistical Science. 2001;16(4):351–367. doi:10.1214/ss/1015346320.
