# Supplementary figures and images for: The role of case proximity in transmission of visceral leishmaniasis in a highly endemic village in Bangladesh
Source: PLoS Negl Trop Dis. 2018 Oct 8;12(10):e0006453. doi: 10.1371/journal.pntd.0006453 (PMC6175508; doi:10.1371/journal.pntd.0006453)

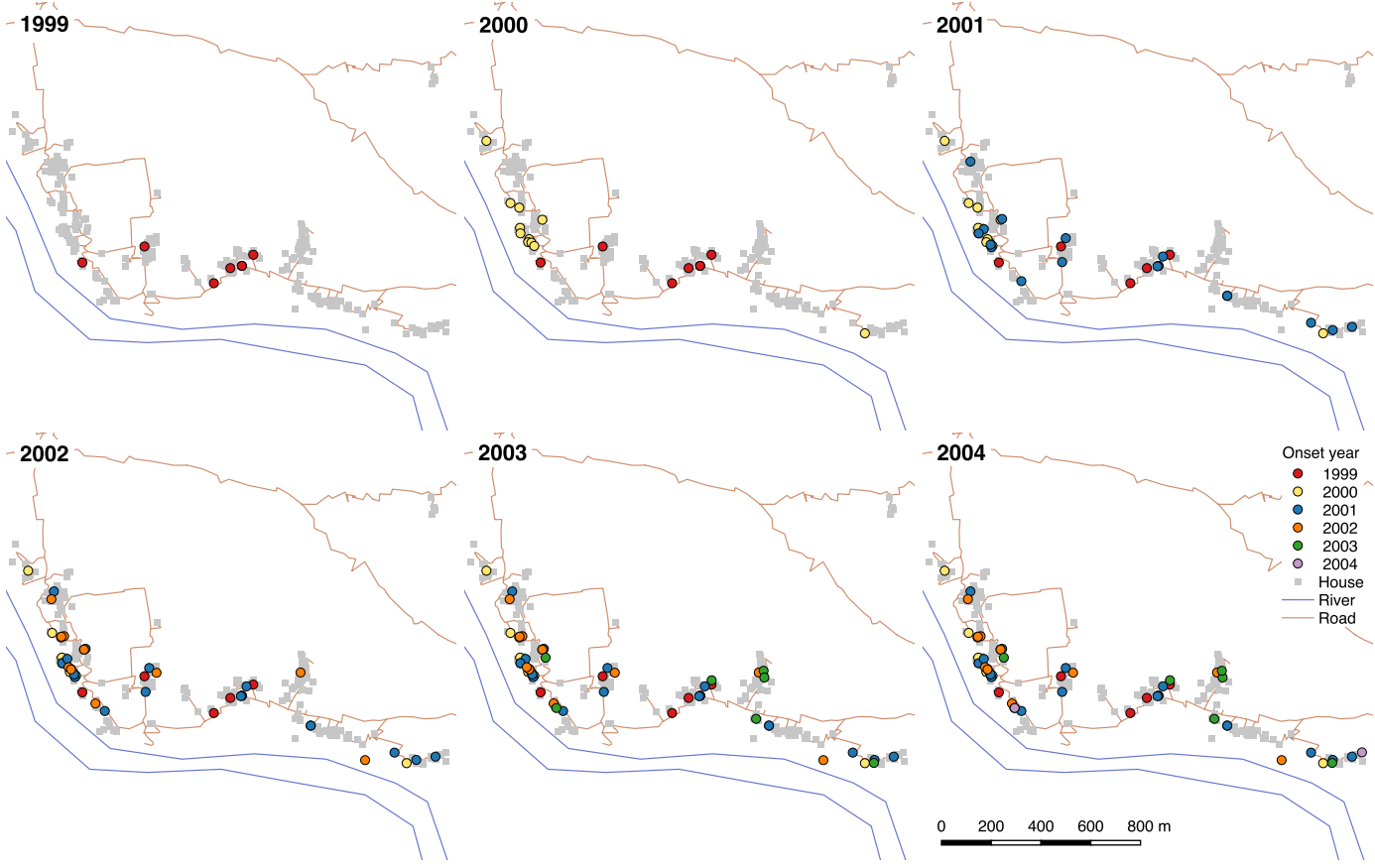

Supplement: S1 Fig — (PDF) [file pntd.0006453.s006.pdf]

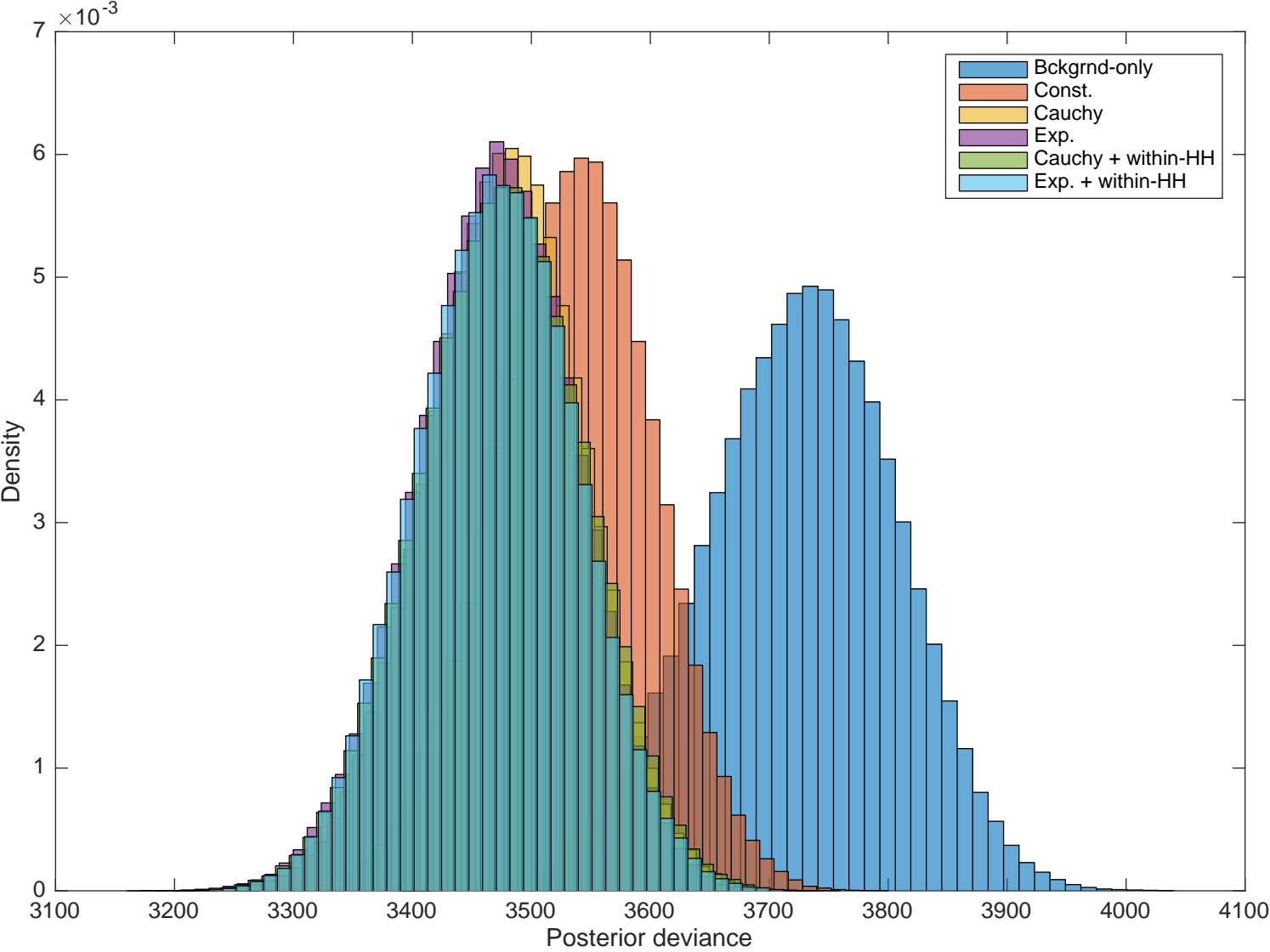

Supplement: S3 Fig — (PDF) [file pntd.0006453.s008.pdf]
