# Supplementary material for: The role of case proximity in transmission of visceral leishmaniasis in a highly endemic village in Bangladesh
Source: PLoS Negl Trop Dis. 2018 Oct 8;12(10):e0006453. doi: 10.1371/journal.pntd.0006453 (PMC6175508; doi:10.1371/journal.pntd.0006453)
Supplement: S2 Fig — (PDF) [file pntd.0006453.s007.pdf]

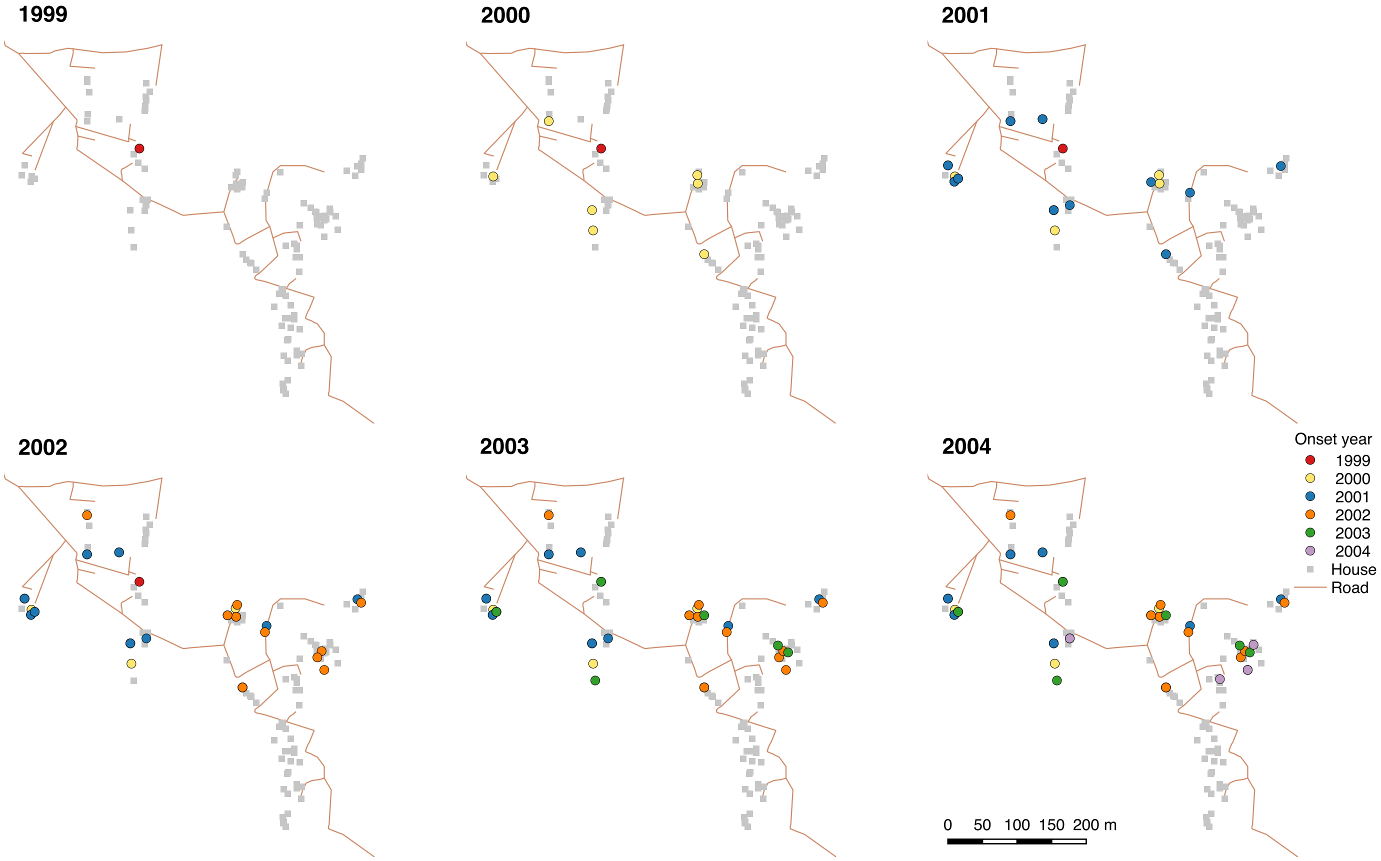
1999
2000
2001
2002
2003
2004
Onset year
1999
2000
2001
2002
2003
2004
House
Road
0 50 100 150 200 m
